# Meta-analysis of *SHANK* Mutations in Autism Spectrum Disorders: A Gradient of Severity in Cognitive Impairments

**DOI:** 10.1371/journal.pgen.1004580

**Published:** 2014-09-04

**Authors:** Claire S. Leblond, Caroline Nava, Anne Polge, Julie Gauthier, Guillaume Huguet, Serge Lumbroso, Fabienne Giuliano, Coline Stordeur, Christel Depienne, Kevin Mouzat, Dalila Pinto, Jennifer Howe, Nathalie Lemière, Christelle M. Durand, Jessica Guibert, Elodie Ey, Roberto Toro, Hugo Peyre, Alexandre Mathieu, Frédérique Amsellem, Maria Rastam, I. Carina Gillberg, Gudrun A. Rappold, Richard Holt, Anthony P. Monaco, Elena Maestrini, Pilar Galan, Delphine Heron, Aurélia Jacquette, Alexandra Afenjar, Agnès Rastetter, Alexis Brice, Françoise Devillard, Brigitte Assouline, Fanny Laffargue, James Lespinasse, Jean Chiesa, François Rivier, Dominique Bonneau, Beatrice Regnault, Diana Zelenika, Marc Delepine, Mark Lathrop, Damien Sanlaville, Caroline Schluth-Bolard, Patrick Edery, Laurence Perrin, Anne Claude Tabet, Michael J. Schmeisser, Tobias M. Boeckers, Mary Coleman, Daisuke Sato, Peter Szatmari, Stephen W. Scherer, Guy A. Rouleau, Catalina Betancur, Marion Leboyer, Christopher Gillberg, Richard Delorme, Thomas Bourgeron

**Affiliations:** 1 Institut Pasteur, Human Genetics and Cognitive Functions Unit, Paris, France; 2 CNRS UMR 3571 Genes, Synapses and Cognition, Institut Pasteur, Paris, France; 3 University Paris Diderot, Sorbonne Paris Cité, Human Genetics and Cognitive Functions, Paris, France; 4 INSERM U975 - CRICM, Institut du cerveau et de la moelle épinière (ICM), CNRS 7225 - CRICM, Hôpital Pitié-Salpêtrière, Paris, France; 5 Sorbonne Universités, UPMC Univ Paris 6, Paris, France; 6 UMR_S 975, Paris, France; 7 Laboratoire de Biochimie, CHU Nîmes, Nîmes, France; 8 Molecular Diagnostic Laboratory and Division of Medical Genetics, CHU Sainte-Justine, Montreal, Quebec, Canada; 9 Department of Medical Genetics, Nice Teaching Hospital, Nice, France; 10 Assistance Publique-Hôpitaux de Paris, Robert Debré Hospital, Department of Child and Adolescent Psychiatry, Paris, France; 11 Departments of Psychiatry, Genetics and Genomic Sciences, Seaver Autism Center, The Mindich Child Health & Development Institute, Icahn School of Medicine at Mount Sinai, New York, New York, United States of America; 12 The Centre for Applied Genomics, The Hospital for Sick Children and the University of Toronto McLaughlin Centre, Toronto, Canada; 13 Laboratoire de Sciences Cognitives et Psycholinguistique, École Normale Supérieure, CNRS, EHESS, Paris, France; 14 FondaMental Foundation, Créteil, France; 15 Department of Clinical Sciences in Lund, Lund University, Lund, Sweden; 16 Gillberg Neuropsychiatry Centre, University of Gothenburg, Gothenburg, Sweden; 17 Department of Molecular Human Genetics, Heidelberg University, Heidelberg, Germany; 18 Wellcome Trust Centre for Human Genetics, University of Oxford, Oxford, United Kingdom; 19 Department of Pharmacy and Biotechnology, University of Bologna, Bologna, Italy; 20 Nutritional Epidemiology Research Unit, INSERM U557, INRA U1125, CNAM, University of Paris 13, CRNH IdF, Bobigny, France; 21 Assistance Publique-Hôpitaux de Paris, Hôpital Pitié-Salpêtrière, Département de Génétique et de Cytogénétique, Unité fonctionnelle de génétique clinique, Paris, France; 22 Centre de Référence “Déficiences intellectuelles de causes rares”, Paris, France and Groupe de Recherche Clinique “Déficience intellectuelle et autisme”, UPMC, Paris, France; 23 Assistance Publique-Hôpitaux de Paris, Hôpital Armand Trousseau, Service de Neuropédiatrie, Paris, France; 24 Département de génétique et procréation, Hôpital Couple-Enfant, Grenoble, France; 25 CADIPA, Centre de Ressources Autisme Rhône-Alpes, Saint Egrève, France; 26 Service de Génétique Médicale, Centre Hospitalier Universitaire Estaing, Clermont-Ferrand, France; 27 UF de Génétique Chromosomique, Centre Hospitalier de Chambéry – Hôtel-dieu, Chambéry, France; 28 UF de Cytogénétique et Génétique Médicale, Hôpital Caremeau, Nîmes, France; 29 CHRU Montpellier, Neuropédiatrie CR Maladies Neuromusculaires, Montpellier, France; 30 U1046, INSERM, Université Montpellier 1 et 2, Montpellier, France; 31 LUNAM Université, INSERM U1083 et CNRS UMR 6214, Angers, France; 32 Centre Hospitalier Universitaire, Département de Biochimie et Génétique, Angers, France; 33 Eukaryote Genotyping Platform, Genopole, Institut Pasteur, Paris, France; 34 Centre National de Génotypage, Evry, France; 35 Hospices Civils de Lyon, CHU de Lyon, Départment de Génétique, Centre de Recherche en Neurosciences de Lyon, CNRS UMR 5292, INSERM U1028, Claude Bernard Lyon I University, Bron, France; 36 Assistance Publique-Hôpitaux de Paris, Hôpital Robert Debré, Genetic department, Cytogenetic Unit, Paris, France; 37 Institute for Anatomy and Cell Biology, Ulm University, Ulm, Germany; 38 Foundation for Autism Research, Sarasota, Florida, United States of America; 39 Montreal Neurological Institute, McGill University, Montreal, Canada; 40 INSERM U1130, Paris, France; 41 CNRS UMR 8246, Paris, France; 42 INSERM U955, Psychiatrie Génétique, Créteil, France; 43 Université Paris Est, Faculté de Médecine, Créteil, France; 44 Assistance Publique-Hôpitaux de Paris, DHU PePSY, Pôle de Psychiatrie et d'Addictologie des Hôpitaux Universitaires Henri Mondor, Créteil, France; 45 Institute of Child Health, University College London, London, United Kingdom; Stanford University School of Medicine, United States of America

## Abstract

*SHANK* genes code for scaffold proteins located at the post-synaptic density of glutamatergic synapses. In neurons, *SHANK2* and *SHANK3* have a positive effect on the induction and maturation of dendritic spines, whereas *SHANK1* induces the enlargement of spine heads. Mutations in *SHANK* genes have been associated with autism spectrum disorders (ASD), but their prevalence and clinical relevance remain to be determined. Here, we performed a new screen and a meta-analysis of *SHANK* copy-number and coding-sequence variants in ASD. Copy-number variants were analyzed in 5,657 patients and 19,163 controls, coding-sequence variants were ascertained in 760 to 2,147 patients and 492 to 1,090 controls (depending on the gene), and, individuals carrying *de novo* or truncating *SHANK* mutations underwent an extensive clinical investigation. Copy-number variants and truncating mutations in *SHANK* genes were present in ∼1% of patients with ASD: mutations in *SHANK1* were rare (0.04%) and present in males with normal IQ and autism; mutations in *SHANK2* were present in 0.17% of patients with ASD and mild intellectual disability; mutations in *SHANK3* were present in 0.69% of patients with ASD and up to 2.12% of the cases with moderate to profound intellectual disability. In summary, mutations of the *SHANK* genes were detected in the whole spectrum of autism with a gradient of severity in cognitive impairment. Given the rare frequency of *SHANK1* and *SHANK2* deleterious mutations, the clinical relevance of these genes remains to be ascertained. In contrast, the frequency and the penetrance of *SHANK3* mutations in individuals with ASD and intellectual disability—more than 1 in 50—warrant its consideration for mutation screening in clinical practice.

## Author Summary

Autism spectrum disorders (ASD) are a heterogeneous group of neurodevelopmental disorders. Mutations altering genes involved in the junction between brain cells have been repeatedly associated in ASD. For example, *SHANK1*, *SHANK2* and *SHANK3* emerged as one family of genes that are associated with ASD. However, little was known about the number of patients carrying these mutations and the clinical outcome. Here, we performed a new genetic screen of *SHANK* mutations and these results were analyzed in combination with those of the literature. In summary, *SHANK* mutations account for ∼1% of patients with ASD and were detected in the whole spectrum of autism with a gradient of severity in cognitive impairment: mutations in *SHANK1* were rare (0.04%) and present in males with normal IQ and autism; mutations in *SHANK2* were present in 0.17% of patients with ASD and mild intellectual disability; mutations in *SHANK3* were present in 0.69% of patients with ASD and up to 2.12% of the cases with moderate to profound intellectual disability. Given the high frequency and impact of *SHANK3* mutations in individuals with ASD and intellectual disability—more than 1 in 50—this gene should be screened for mutations in clinical practice.

## Introduction

Autism spectrum disorders (ASD) are characterized by impairments in reciprocal social communication and stereotyped behaviors. There is strong evidence of the involvement of different forms of genetic variations in ASD [1], [2]. In particular, chromosomal rearrangements, rare *de novo* copy-number variants and *de novo* coding-sequence variants may account for more than 20% of the cases [1], [2]. These events have implicated more than 100 genes [3], but each gene or genomic alteration often accounts for less than 1% of the cases. Many of the genes associated with the disorder are involved in the development or functioning of neuronal circuits [4]. In particular, mutations in genes coding for synaptic cell adhesion molecules and scaffold proteins — such as neuroligins, neurexins and *SHANK* — have been repeatedly reported in individuals with ASD [5]–[10]. These proteins play a crucial role in the formation and stabilization of synapses [11], [12]. The synapse has therefore emerged as a common target for the different genetic mutations that affect chromatin remodeling, synaptic translation, formation and functioning [4].

Here, we focused on the three *SHANK* genes, which code for large synaptic scaffold proteins of the post-synaptic density [12]. Deletions, duplications and coding mutations in the *SHANK* genes have been recurrently reported in patients with ASD [6], [8]–[10], [13]–[20]. *SHANK3* haploinsufficiency has been identified in more than 900 patients affected with chromosome 22q13 deletion syndrome, known as Phelan–McDermid syndrome [15]. The genomic rearrangements observed in these patients are diverse ranging from simple 22q13 deletions (72%), ring chromosomes (14%), unbalanced translocations (7%) to interstitial deletions (9%), all resulting in haploinsufficiency of the *SHANK3* gene [21].

The majority of these patients have neonatal hypotonia, moderate to severe intellectual disability (ID), absent to severely delayed speech, and minor dysmorphic features [15]. In more than 80% of the cases, autism or autistic-like behavior is present [22]. *De novo* or truncating mutations in *SHANK3* have also been observed in individuals with ASD [6], [13], [16], [17]. Few studies have explored *SHANK1* and *SHANK2* in ASD, but all have led to the conclusion that deleterious mutations in these genes contribute to the disorder [14], [18], [19].

Mice lacking any of the SHANK proteins display phenotypes relevant to ASD [23]. *Shank1* knock-out mice show increased anxiety, decreased vocal communication, decreased locomotion and remarkably, enhanced working memory, but decreased long-term memory [24], [25]. *Shank2* knock-out mice show hyperactivity, increased anxiety, repetitive grooming, and abnormalities in vocal and social behaviors [26], [27]. *Shank3* knock-out mice show self-injurious repetitive grooming, and deficits in social interaction and communication [28]–[30].

While there is increasing evidence of an association between *SHANK* genes and ASD, *SHANK* mutations are considered to affect only a limited number of patients and as a consequence, these genes are not routinely sequenced in clinical practice. In addition, sequence gaps and annotation errors of *SHANK2* and *SHANK3* in the human genome assembly (hg19) have led to incorrect interpretations of sequencing results obtained in patients [15]. Finally, the clinical impact of the mutations in the *SHANK* genes is still largely unknown.

Our hypothesis was that mutations in *SHANK* genes might be more frequent in patients with ASD than previously suggested, and that each gene might be associated with specific clinical profiles. To conduct this study, we first corrected the reference sequence of *SHANK2* and *SHANK3* (Table S1, Figure S1 & S2 and Text S1: Supplementary Methods). We then analyzed a large number of individuals with ASD for *SHANK* copy-number variants and coding-sequence variants and combined these results with those reported in the literature. Finally, we performed an extended clinical investigation in all patients carrying *de novo* or truncating *SHANK* mutations.

## Results

### Cohorts used for the meta-analysis of *SHANK* mutations

**Figure 1:**
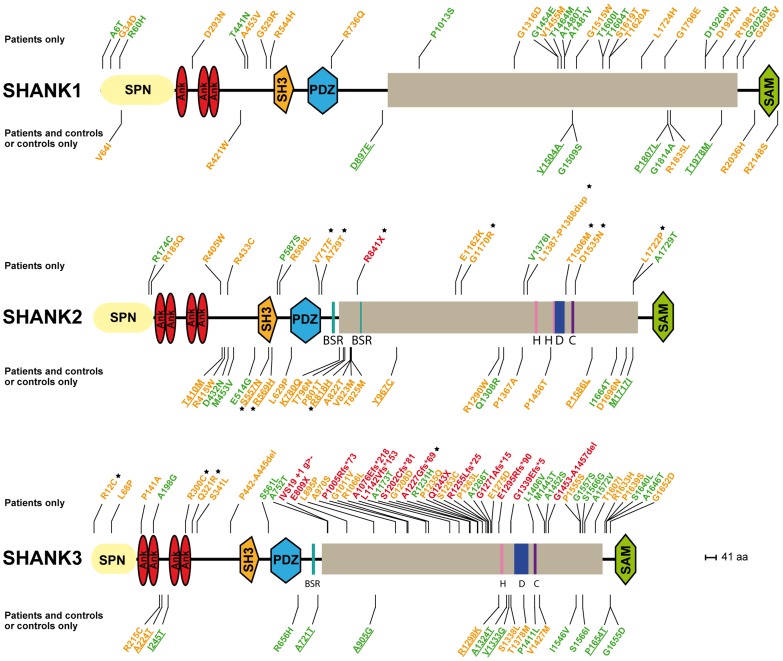
*SHANK* variants in patients with ASD and controls. Coding-sequence variants identified only in patients with ASD (upper panel), shared by patients and controls (lower panel and underlined), and present only in controls (lower panel). Truncating variants are indicated in red. The variants predicted as deleterious or benign are indicated in orange and green, respectively. Coding-sequence variants with a proven *in vitro* functional impact are indicated with black stars. Conserved domains are represented in color: SPN (yellow), Ankyrin (red), SH3 (orange), PDZ (blue) and SAM (green).

**Figure 2:**
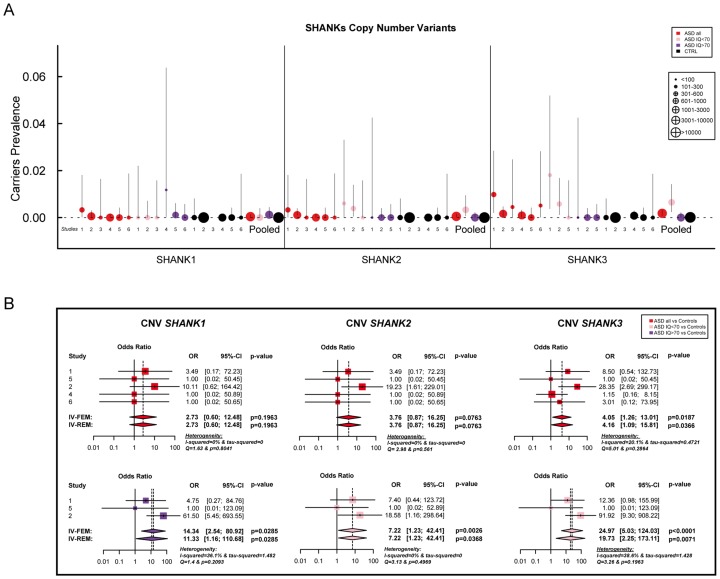
Prevalence and meta-analysis of copy number variant studies in ASD. A. The prevalence and the confidence interval from a set of single copy number variant studies and the pooled prevalence and the confidence interval of the meta-analysis. The prevalence is indicated by circles in red, pink, purple and black for “ASD all” (all ASD patients), “ASD IQ<70” (patients with ID; IQ<70), “ASD IQ>70” (patients with IQ in the normal range), and “CTRL” (controls), respectively. The plotted circles are proportional to the corresponding sample size. B. Meta-analysis of the copy number variants altering *SHANK* genes. For each study, the Odds ratio and confidence interval are given. Each meta-analysis is calculated using inverse variance method for fixed (IV-FEM) and random effects (IV-REM). The statistics measuring heterogeneity (Q, I^2^ and Tau^2^) are indicated. The number under the scatter plot correspond to independent studies: 1 = “[The Paris cohort: this study+Durand et al. 2007 [6]; Sato et al. 2012 [19]; Leblond et al. 2012 [18]]”, 2 = “[Moessner et al. (2007) [13]; Marshall et al. (2008) [52]; Pinto et al. (2010) [8]; Berkel et al. (2010) [14]; Sato et al. (2012) [19]]”, 3 = “Bremer et al. (2010) [53]”, 4 = “Glessner et al. (2009) [34]”, 5 = “Sanders et al. (2011) [9]”, and 6 = “Sebat et al. (2007) [51]”. IV, Inverse Variance; FEM, Fixed Effect Method; REM, Random Effect Method; OR, Odds Ratio; CI, Confidence Interval; IQ, Intellectual Quotient; CNV, Copy Number Variant.

**Figure 3:**
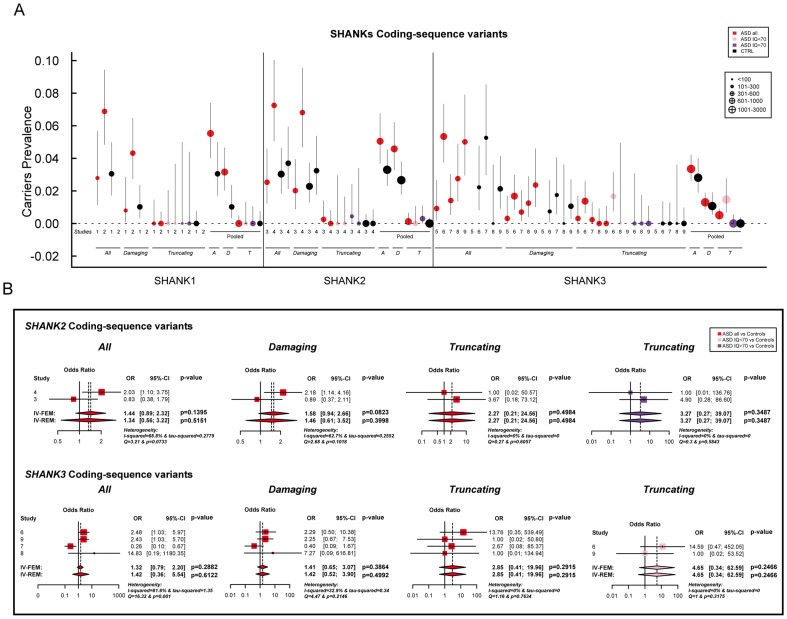
Prevalence and meta-analysis of coding-sequence variant studies in ASD. A. The prevalence and the confidence interval from a set of single coding-sequence variant studies, and the pooled prevalence and the confidence interval of the meta-analysis. The prevalence is indicated by circles in red, pink, purple and black for “ASD all” (all ASD patients), “ASD IQ<70” (patients with ID; IQ<70), “ASD IQ>70” (patients with normal IQ), and “CTRL” (controls), respectively. Three categories are used to study the prevalence of coding-sequence variants in ASD and controls: all or “A” (all mutation), Damaging or “D” (damaging missense mutation; score obtained from polyphen-2), and Truncating or “T” (mutation altering SHANK protein). The plotted circles are proportional to the corresponding sample size. B. Meta-analysis of coding-sequence variant studies altering *SHANK* genes. For each study, the Odds ratio and confidence interval is given. Each meta-analysis is calculated using inverse variance method for fixed (IV-FEM) and random effects (IV-REM). The statistics measuring heterogeneity (Q, I^2^ and Tau^2^) are indicated. The number under the scatter plot correspond to independent studies: 1 = “This study”, 2 = “ Sato et al. (2012) [19]”, 3 = “Berkel et al. (2010) [14]”, 4 = “Leblond et al. (2012) [18]”, 5 = “Boccuto et al. (2012) [17]”, and 6 = “[This Study and Durand et al. 2007 [6]]”, 7 = “[Gauthier et al. (2009–2010) [16], [47]]”, 8 = “Moessner et al. (2007) [13]”, 9 = “Schaff et al. (2011) [35]”. IV, Inverse Variance; FEM, Fixed Effect Method; REM, Random Effect Method; OR, Odds Ratio; CI, Confidence Interval; IQ, Intellectual Quotient; CNV, Copy Number Variant.

**Figure 4:**
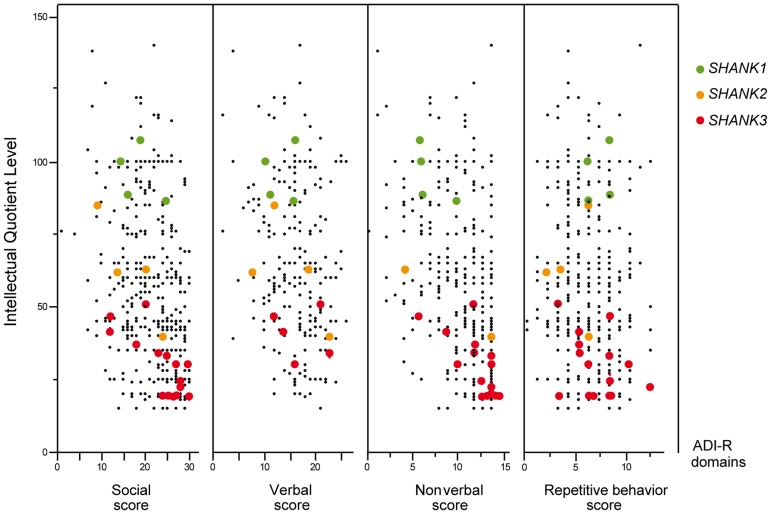
Scatter plots of the intellectual quotient and the Autism Diagnostic Interview-Revised (ADI-R) scores of the patients with ASD screened for *SHANK1-3* mutations. Mutations in *SHANK1-3* are associated with a gradient of severity in cognitive impairment. *SHANK1* mutations were reported in patients without ID (green dots). *SHANK2* mutations were reported in patients with mild ID (orange dots). *SHANK3* mutations were found in patients with moderate to severe deficit (red dots). Black dots correspond to the patients enrolled in the PARIS cohort screened for deleterious *SHANK1-3* mutations (n = 498). In addition to the PARIS cohort [6], [8], [18], three patients with a *SHANK1* deletion [19] and two patients with a *SHANK2* deletion [14] were included in the scatter plot. A high score of the ADI-R is associated with a more severe profile. The threshold of the “Social”, “Verbal”, “Non-Verbal” and “Repetitive Behavior” Scores are 10, 8, 7 and 3, respectively.

We performed a meta-analysis of copy-number variants and coding sequence variants in all *SHANK* genes (Figure 1). This meta-analysis included the published data from 14 studies in addition to a new screening of SHANK copy-number variants and coding sequence variants. The number of individuals tested and the result of the meta-analysis are reported in the Table 1, Figures 2 and 3 and Table S2, S3, S4, S5, S6. In addition to the results from the literature, we performed a new copy-number variants analysis of 46 additional cases with ASD and 454 matched controls. We also performed a mutation screening of all *SHANK1* exons in 743 independent individuals including 251 cases with ASD and 492 controls. Finally, we added 429 new independent cases with ASD and 80 new independent controls to our original screening of coding-sequence variants of *SHANK3* [6]. When possible, the patients carrying truncating mutations altering *SHANK* genes underwent further clinical investigations (Table 2 & S7). The meta-analysis of the frequency of CNVs and coding-sequence variants altering *SHANK* genes in patients with ASD and in controls were also performed using IQ as a co-variable (Figure 4).

**Table 1:** Prevalence of *SHANK* rare coding-sequence and copy-number variants in patients with ASD and controls.

	Number of studies	Number of reported carriers of rare *SHANK* variants		Prevalence of carriers of rare *SHANK* variants (%)		Fisher's exact test		Meta-analysis - Inverse variance method (FEM)		Meta-analysis - Inverse variance method (REM)	
	Total	ASD	Controls	ASD	Controls	Odds ratio	*P*-value	Odds ratio	*P*-value	Odds ratio	*P*-value
***SHANK1***											
**Coding-sequence variants** a	2^b^										
All		42/760	15/492	5.53 [4.01–7.4]	3.05 [1.72–4.98]	1.86 [1.00–3.65]	0.051	-	-	-	-
Damaging missense		24/760	5/492	3.16 [2.03–4.66]	1.02 [0.33–2.36]	3.17 [1.18–10.72]	**0.012**	-	-	-	-
Truncating mutation		0/760	0/492	0.00 [0–0.48]	0.00 [0–0.75]	0 [0-∞]	1	-	-	-	-
**Copy-number variants**	6	2/5 657	0/19 163	0.04 [0–0.13]	0.00 [0–0.02]	∞ [0.64-∞]	0.052	2.73 [0.60–12.48]	0.19	2.73 [0.60–12.48]	0.19
***SHANK2***											
**Coding-sequence variants**	2										
All		43/851	36/1 090	5.05 [3.68–6.75]	3.30 [2.32–4.54]	1.56 [0.97–2.52]	0.064	1.44 [0.89–2.32]	0.13	1.34 [0.56–3.22]	0.51
Damaging missense		39/851	29/1 090	4.58 [3.28–6.21]	2.66 [1.79–3.8]	1.76 [1.05–2.97]	**0.025**	1.58 [0.94–2.66]	0.082	1.46 [0.61–3.52]	0.39
Truncating mutation		1/851	0/1 090	0.12 [0–0.65]	0.00 [0–0,34]	∞ [0.033-∞]	0.44	2.27 [0.21–24.56]	0.49	2.27 [0.21–24.56]	0.49
**Copy-number variants**	6	3/5 657	0/19 163	0.05 [0.01–0.15]	0.00 [0.02]	∞ [1.40-∞]	**0.012**	3.76 [0.87–16.25]	0.076	3.76 [0.87–16.25]	0.076
***SHANK3***											
**Coding-sequence variants**	5										
All		72/2 147	29/1 031	3.35 [2.63–4.20]	2.81 [1.89–4.01]	1.20 [0.76–1.93]	0.45	1.32 [0.79–2.20]	0.29	1.42 [0.36–5.54]	0.61
Damaging missense		28/2 147	11/1 031	1.30 [0.87–1.88]	1.07 [0.53–1.9]	1.22[0.59–2.74]	0.73	1.41 [0.65–3.07]	0.38	1.42 [0.51–3.90]	0.50
Truncating mutation		11/2 147	0/1 031	0.51 [0.26–0.91]	0.00 [0–0.36]	∞ [1.21-∞]	**0.020**	2.85 [0.41–19.96]	0.29	2.85 [0.41–19.96]	0.29
**Copy-number variants**	6	10/5 657	2/19 163^c^	0.18 [0.08–0.32]	0.01 [0–0.04]	16.96 [3.61–159.14]	**0.000016**	4.05 [1.26–13.01]	**0.019**	4.16 [1.09–15.81]	**0.036**

a All truncating *SHANK* variants were *de novo* (for three, the DNA of one parent was not available). In the damaging missense category, two *SHANK3* (P141A & Q321R) were *de novo*.

b For *SHANK1*, there are two studies (Sato et al. (2012) [19] and this study), but Sato et al. (2012) [19] did not screen for all *SHANK1* exons in the controls. Therefore these controls were not included here.

c The two *SHANK3* deletions reported by Glessner et al. (2009) [34] in control subjects have not been validated and should be interpreted with caution. The frequencies of *SHANK* mutations have been calculated including only unrelated cases and controls. FEM, Fixed Effects Model; REM, Random Effects Model. After Bonferroni correction for 12 tests (significant threshold corrected α-value = 0.0042), only the *SHANK3* copy-number variant association remains significant. The power achieved to observe the statistical difference between patients and controls for *SHANK1* and *SHANK2* damaging missense variants was 69% and 59%.

**Table 2:** Clinical characteristics of the patients carrying *de novo SHANK2* and *SHANK3* mutations.

	Patients					
SHANK2	SK0217-003	6319_3	AU038_3	RDB_30769	AUL_001	Wischmeijer et al. 2010
	Pinto et al. 2010	Pinto et al. 2010	Leblond et al. 2012	This study	This study	Wischmeijer et al. 2010
**Sex**	M	M	M	M	M	F
**Mutation**	CNV_del (66 kb)	CNV_del (68 kb)	CNV_del (421 kb)	CNV_del (1.8 Mb)	Translocation	CNV_del (3.4 Mb)
	Loss of exon 6 & 7	Loss of exon 14 & 15	Loss of exon 5 to 16	del_11q13.3q13.4 (all *SHANK2* exons)	t(1;7;11)(p35;q33;q12)dn; breakpoint in *SHANK2 (intron 14)*	del_11q13.2q13.4 (all *SHANK2* exons)
	*De novo*	*De novo*	*De novo*	*De novo*	*De novo*	*De novo*
**Diagnosis and cognitive development**	Autism	Autism	Autism	Autism	Autism	Autism
	Early DD (mild motor & language delay)	Early DD (mild motor & language delay)	Early DD (mild motor & language delay)	Global DD	Global DD	Global DD
	Mild ID/verbal	Mild ID/verbal	Mild ID/verbal	Severe ID/non verbal	Severe ID/non verbal	Severe ID/non verbal
		Neonatal hypotonia			Neonatal hypotonia	Neonatal hypotonia
**Dismorphic signs**	Clinodactyly (5^th^ fingers)	Large ears	Clinodactyly (5^th^ fingers)	Clinodactyly (5^th^ fingers)	Clinodactyly (5th fingers)	Deep-set eyes & epicanthus
	Large ears	Pointed chin	Deep-set eyes	Deep-set eyes	Deep-set eyes & epicanthus	Long eyelashes & ptosis
	Long eyelashes	Wide nasal bridge	Large ears	Strabismus & ptosis	Large ears	Wide nasal bridge
	Wide nasal bridge	Retrognathia	Pointed chin	Large ears	Wide nasal bridge	Thin upper lip
			Retrognathia	Retrognathia	Long eyelashes	Retrognathia
			Wide nasal bridge	Wide nasal bridge	Retrognathia	Clinodactyly (5^th^ fingers) & syndactylia (2^nd^–3^rd^)
			Thin upper lip	Thin upper lip		
**Motor signs**		Oral dyspraxia	Oral dyspraxia	Oral dyspraxia		Oral dyspraxia
		Slight hypotonia	Slight hypotonia	Slight hypotonia		Slight hypotonia
		Signs of cerebellar dysfunction (dysmetry & dysdiadochokinesis)	Signs of cerebellar dysfunction (dysmetry & dysdiadochokinesis)	Signs of cerebellar dysfunction (dysmetry & dysdiadochokinesis)		

	Patients						
SHANK3	AUN_001	AUN_002	AUN_003	AUN_004	AU285_4	AU921_3	AUN_005
	This study	This study	This study	This study	This study	This study	This study
**Sex**	F	M	F	M	M	M	M
	CSV	CSV	CSV	CSV	CSV	CSV	CSV
	P1005Rfs*73	G1339Efs*5	E809X	S1202Cfs*81	Q1243X	L1142Vfs*153	R1255LfsX25
	Unknown^a^	Unknown^a^	*De novo*	*De novo*	*De novo*	*De novo*	*De novo*
**Diagnosis and cognitive development**	Autism	Autism	Autism	Autism	Autism	Autism	Autism
	Global DD	Global DD	Global DD	Global DD	Global DD	Global DD	Global DD
	Severe ID/non verbal	Severe ID/non verbal	Severe ID/verbal	Moderate ID/verbal	Severe ID/non verbal	Severe ID/non verbal	Moderate ID/verbal
	Early regression	Late regression	Early regression	Early regression		Early regression	
**Dismorphic signs**	Large ears	Long face	Large detached ears		Large ears	Large ears	Large ears
	Long eyelashes	Long eyelashes	Bulbous nose		Long eyelashes	Puffy eyelids	Wide nasal bridge
	Wide nasal bridge	Deep-set eyes	Pointed chin		Deep-set eyes	Deep-set eyes	Bulbous nose
	Smooth philtrum	Smooth philtrum	Retrognathia		Wide nasal bridge	Wide nasal bridge	Fleshy hands
		Prognathia			Strabismus	Bulbous nose	
		Dysplastic toe nails			Dental malocclusion	Pointed chin	
**Motor signs**	Hypotonia	Hypotonia	Hypotonia		Hypotonia		Hypotonia & microcephaly (-2.5SD)
		Ataxia & dysmetria					
**Others**	Cycling vomiting	Gastroesophageal reflux	Cholesteatoma	Migraines	Gastroesophageal reflux	Scoliosis	Severe feeding difficulties, Gastroesophageal reflux
		Epilepsy-GTCS (AAO: 8 y, Resistance)				Epilepsy-GTCS (AAO: 10 y, Responder)	Epilepsy-Absence seizures (AAO: unk)
		Chewing non-food items				Severe stereotypies	

a Mutations not present in the mother and father not tested (DNA unavailable). M, Male; F, Female; CNV, Copy Number Variant; del, deletion; CSV, Coding-Sequence Variant; DD, developmental delay; ID, intellectual disability; GTCS, Generalized Tonic-Clonic Seizures; AAO, Age at onset; y, years; SD, Standard Deviation.

### *SHANK1* in ASD

Altogether, our new screening of 306 patients with ASD and 454 controls and the previously published copy-number variants studies (Table S3 & S5) showed that deletions disrupting *SHANK1* were detected in 0.04% (n = 2/5 657) of patients with ASD and were never found in 19 163 controls (Meta-analysis – Inverse variance method - fixed effect model *P* = 0.19, OR = 2.73, 95% CI = 0.60–12.48) (Table 1 and Figure 2). The two independent families with *SHANK1* deletions were reported by Sato *et al.* (2012) [19]. A *de novo* deletion of 63.4 kb altering both the synaptotagmin-3 gene (*SYT3*) and *SHANK1* was detected in a Swedish male with normal IQ and ASD [19]. An inherited exonic deletion of 63.8 kb altering both *SHANK1* and *CLEC11A* segregated in a four-generation Canadian family in which male carriers—but not female carriers—have ASD [19]. No *SHANK1* duplications were found.

We screened a sample of 251 patients and 492 controls for *SHANK1* coding exons. As for the *SHANK1* mutation screening by Sato et al. (2012), no *de novo* truncating mutation sequences were identified. Based on the two cohorts of 760 patients with ASD and 492 controls (Table 1, Figure 1 & 3, Tables S4, S5, S6, S7 & S9), rare inherited coding-sequence variants predicted as damaging were, however, more frequent in patients with ASD than in controls (3.16% in ASD, 1.02% in controls, Fisher's exact test two-sided, P = 0.012, OR = 3.17, 95% CI = 1.18–10.72). The rare variants observed in patients with ASD were not observed in an additional sample of 500 control chromosomes.

The 4 males with *SHANK1* deletions were diagnosed with ASD and an IQ in the normal range (mean IQ = 107) and good verbal ability without significant language delay [19] (Figure 4). Interestingly, sex differences might modulate the phenotype since females carrying an inherited *SHANK1* deletion exhibited anxiety and shyness, but did not fulfill criteria for ASD [19].

### *SHANK2* in ASD

*SHANK2* deletions were found in 0.05% (n = 3/5 657) of patients with ASD, and never in controls (n = 0/19 163; Meta-analysis – Inverse variance method - fixed effect model, P = 0.076, OR = 3.76, 95% CI = 0.87–16.25) (Table 1, S3 & S5, and Figure 2). All deletions were *de novo* and disrupted coding exons. No *SHANK2* duplications were reported. We identified two patients with a *SHANK2 de novo* deletion (Table 2). For patient AUL_001, the breakpoints were previously sequenced using whole genome sequencing [31] (Table 2, Figure S4 and Text S1). The second patient (RDB_30769) carried a *de novo* deletion of 1.8 Mb encompassing *SHANK2*. These two patients were not included in the calculation of the prevalence since they were not part of our cohort screened for copy-number variants. They were identified during clinical screening and the exact number of patients with ASD investigated was not available.

Based on the mutation screening of Berkel et al. (2010) and Leblond et al. (2012), the prevalence of truncating *SHANK2* coding-sequence variants was 0.12% in patients with ASD (n = 1/851), and such variants were not found in any of the 1 090 controls (Figure 1 and 3, Table 1, Table S4, S5, S6, S7 & S10). This prevalence is similar to the one reported in the four large-scale studies in ASD using exome sequencing [32] (*de novo* or truncating *SHANK2* mutation 1/965, 0.10%). Finally, we observed rare coding-sequence variants predicted as damaging in 4.58% of the patients with ASD compared with 2.66% of the controls (Fisher's exact test two-sided, P = 0.025, OR = 1.76, 95% CI = 1.05–2.97).

The individuals carrying a *SHANK2 de novo* deletion were diagnosed with autistic disorder or pervasive developmental disorder not otherwise specified (PDD-NOS) in combination with mild to moderate ID (mean IQ = 62±17) (Figure 4). They displayed early signs of developmental delay, mild motor delay and significant language delay. They also displayed minor signs of dysmorphism (broad nasal bridge, thin upper lip, pointed chin, clinodactyly) and abnormal neurological examination (Table 2). Specifically, cases 6319-3 and AU038-3 had mild axial hypotonia, oral dyspraxia and minor signs or cerebellar dysfunction (including dysmetry and dysdiadochokinesis). These clinical signs are unspecific, but were also reported in patients with ASD with more complex chromosomal rearrangements encompassing *SHANK2* [33]. The individual carrying the *de novo* truncating mutation R841X (SK 0441-003) had a normal IQ and diagnosed with ASD without any developmental delays or dysmorphic features.

### *SHANK3* in ASD

In our screening of 306 patients with ASD, we identified one patient (AU029) carrying a *de novo SHANK3* deletion of 1.5 Mb (Figure S4). Altogether, *SHANK3* deletions were detected in 0.18% of patients with ASD (n = 10/5 657) and in 0.01% of controls (n = 2/19 163) (Meta-analysis – Inverse variance method - fixed effect model, P = 0.019, OR = 4.05, 95% CI = 1.26–13.01) (Figure 2, Figure S4, Table 1 and Table S3, S4, S5, S6, S7). Deletions of *SHANK3* have not been reported in controls before, so the two deletions reported by Glessner *et al.* (2009) [34], which have not been validated, should be interpreted with great caution. In three families from France and Canada, the *SHANK3* deletions originated from a balanced translocation present in a healthy parent [6], [13]. Interestingly, in two families, a sibling carried the reciprocal *SHANK3* duplication. In the French family, the elder brother carrying the *SHANK3* duplication was diagnosed with Asperger syndrome [6]. In the Canadian family, the elder sister carrying the *SHANK3* duplication was diagnosed with attention-deficit/hyperactivity disorder (ADHD) and developmental delay [13]. In a screen of 160 additional patients with ASD and ID using Multiplex Ligation-dependent Probe Amplification (MLPA) analysis, we observed two patients carrying a *de novo* deletion altering *SHANK3* (Figure S4 and Text S1: Supplementary Methods). For patient AUN_006, the deletion breakpoint is located within intron 8 of *SHANK3* and leads to the loss of *SHANK3* (exons 9 to 22), *ACR* and *RABL2B*. For the second patient AUN_007, the deletion covers the exon 22 of *SHANK3* and exons 1 to 3 of *ACR*.

We screened 429 patients with ASD for all coding exons of *SHANK3* and found 8 patients carrying heterozygous truncating mutations (Figure 1 and 3, Figure S5, Table 1, Table S4, S5, S6, S7 and Table S11), including 6 that appeared *de novo* in the probands. For the remaining two, the mothers were not carriers, but the DNA of the fathers were not available. When all mutation screenings were included, truncating *SHANK3* coding-sequence variants were found in 0.51% of the patients (n = 11/2 147) and were not found in 1031 controls (meta-analysis – inverse variance method - fixed effect model, P = 0.29, OR = 2.85, 95% CI = 0.41–19.96) (Table 1, S5 & S11, and Figure 1 & 3) [6], [13], [16], [17], [35]. We observed an enrichment of truncating mutations in exon 21a of *SHANK3*. We therefore screened an additional sample of 138 cases with ASD for exon 21a and identified a novel *de novo* stop mutation (Q1243X) in one boy with autism and moderate ID (Table 2 & Figure S5).

Individuals with *SHANK3* truncating mutations displayed autism with moderate to severe/profound ID (mean IQ: 31±8) (Figure 4). The individuals carrying *SHANK3* deletions had also manifestations of the Phelan-McDermid syndrome [15]. For example, the boy carrying the L1142Vfs*153 mutation was non-verbal, showed a global developmental delay with neonatal hypotonia and typical dysmorphic features of Phelan-McDermid syndrome, including wide nasal bridge, pointed chin, deep-set eyes, flat mid-face, large ears, long eyelashes, bulbous nose, and high-arched palate (Table 2). He also developed generalized epilepsy at the age of 10 years, which was characterized by intolerance and resistance to variety of anticonvulsant medications. By contrast, the boy carrying the *de novo* truncating mutation S1202Cfs*81 was verbal and had moderate ID. Although reduced in quality by the opposition of the patient, the clinical examination was considered in the normal range with no significant dysmorphic features. Thus, the phenotypic variability of the Phelan-McDermid syndrome, which was considered to result from the wide range of deletion sizes, was also observed for individuals carrying *SHANK3 de novo* or truncating mutations.

## Discussion

Mutations of the *SHANK* genes were detected in the whole spectrum of ASD with a gradient of severity in cognitive impairment. *SHANK1* mutations were detected in individuals with ASD and normal IQ, *SHANK2* mutations were found in cases with ASD and mild ID, and *SHANK3* mutations were mainly found in individuals with ASD combined with moderate to severe ID. In the whole spectrum of ASD, we estimated that 0.04%, 0.17% and 0.69% of cases with ASD had heterozygous truncating mutations in *SHANK1*, *SHANK2* or *SHANK3*, respectively (Table 1). Recent exome sequencing studies only reported one *de novo SHANK2* mutation out of 965 patients [32], [36], [37] and no truncating coding-sequence variation within *SHANK1* and *SHANK3*. In contrast, we report 0.51% of cases with ASD carrying truncating coding-sequence variations in *SHANK3*. This difference could be explained by the very low sequencing coverage of *SHANK3* using whole-exome sequencing technology leading to a low power of detection of *SHANK3* mutations. This coverage issue of *SHANK3* was indeed observed by other groups [38]. Interestingly, regions with low coverage of *SHANK3* correlate with high percentage of GC; and the majority of the mutations detected in our study were located in the exonic region of *SHANK3* showing a very low coverage (Figure S3).

The prevalence of each *SHANK* mutations appeared to be different when the severity of cognitive impairment was considered (Figure 4, Table 3 and Table S6). This was particularly relevant for *SHANK3* in individuals with ASD and ID. The prevalence of *de novo* or truncating *SHANK3* mutations in these patients was 2.12% (copy-number variants: 6/917 patients with IQ<70; prevalence = 0.65%; coding-sequence variants: 9/611 patients with IQ<70; prevalence = 1.47%), 0% in patients with ASD without ID and 0.01% in controls. Our prevalence of *SHANK3* deletions in patients with ASD and ID is similar to that reported by Cooper *et al.* (2011) in a large sample of 1 379 patients with autism and developmental delay (0.87%) [10]. Altogether, in addition to the large deletions observed in Phelan-McDermid syndrome, mutations of *SHANK3* account for more than 1 out of 50 cases diagnosed with the combination of ASD and ID. Detection of such mutations should therefore be considered in clinical practice. This clinical screening should: (i) improve the quality of genetic counseling of ASD and ID for patients and their family relatives; (ii) increase our understanding of the clinical features associated with SHANK mutations together with the developmental trajectories of the patients [39], (ii) enable the development of a large number of independent induced pluripotent stem cells (iPSC) carrying SHANK mutations [40], (iii) set the ground for future large scale clinical trials targeting these synaptic defects [41].

**Table 3:** Summary of the SHANK protein functions and of the main findings obtained for patients with ASD.

		*SHANK1*	*SHANK2*	*SHANK3*
**DNA**	chromosome	19q13.3	11q13.3	22q13.3
	damaging mutations in Controls	1.02%	2.66%	1.07%
	truncating mutations in Controls	0%	0%	0%
**RNA & Proteins**	mRNA localization in neurons	soma and dendrites (hippocampal & Purkinje cells)	soma and dendrites (Purkinje cells)	soma and dendrites (hippocampal neurons)
	expression pattern	high in cortex	broad in brain (cerebellar Purkinje cells)	high in striatum (cerebellar granule cells)
**Synapses**	localization	glutamatergic synapses	glutamatergic synapses	glutamatergic synapses
	expression dynamics	3^rd^ Shank at the synapse	1^st^ Shank at the synapse	2^nd^ Shank at the synapse
	effect loss	decrease in GKAP & Homer	increase in NMDAR NR1	decrease in NMDAR NR1 and AMPAR
	zinc dependence	independent	dependent	dependent
**Spines**	effect of loss	decreased size of spine heads	decreased number of mature spines	decreased number of mature spines
	effect of gain		increased number of mature spines	increased number of mature spines
	effect of mutation in ASD		reduction of synaptic density affect spine induction & morphology	reduction of synaptic density affect spine induction & morphology
**Synaptic currents**	effect of loss	normal NMDA and AMPA	increase/decrease NMDA*	decrease NMDA and AMPA
**Mouse behavior**	social interactions	reduced	reduced	reduced
	vocal behaviors	abnormal	abnormal	abnormal
	activity	reduced	increased	reduced
	stereotypies		increased	increased
	learning	enhanced (but reduced memory)	reduced	reduced
**Truncating mutations**	ASD	0.04%	0.17%	0.69%
	ASD (IQ>70)	0.12%	0.30%	0%
	ASD (IQ≤70)	0%	0.33%	2.12%
**IQ**	ASD	95±11	62±17	30±8
**Penetrance**	males	high	high	high
	females	incomplete	not reported	high

The frequency of mutation in patients and control individuals was calculated from the total cohort (Table 1). The frequency of mutation in patients with normal IQ (IQ>70) and low IQ (IQ<70) were calculated for the patients with available IQ scores (copy-number variants for all SHANK: nASD with IQ>70 = 1 638 & nASD with IQ<70 = 917; SHANK1 coding-sequence variants: nASD with IQ>70 = 354 and nASD with IQ<70 = 278; SHANK2 coding-sequence variants: nASD with IQ>70 = 335 & nASD with IQ<70 = 344; SHANK3 coding-sequence variants: nASD with IQ>70 = 667 & nASD with IQ<70 = 611). The mean IQ and standard deviation was given only for patients carrying truncating or *de novo* mutations. The black star indicates that Schmeisser et al. (2012) [21] found an increase in NMDA currents, while Won et al. (2012) [22] found a decrease in NMDA currents in two independent SHANK2 knock-out mice.

Contrary to the *de novo SHANK* mutations, the role of the inherited sequence variants remains difficult to ascertain. Our study provides some insights regarding this issue. There is a trend for more *SHANK1* (unadjusted P = 0.012) and *SHANK2* (unadjusted P = 0.025) inherited deleterious mutations in patients with ASD than in controls (Table 1). However, these associations do not survive Bonferroni correction for multiple testing. Despite our meta-analysis that includes several mutations screening, we were underpowered to detect associations with low effect size (Table S12). The power to detect an odds ratio of 1.5 (two-sided) for *SHANK1*, *SHANK2* and *SHANK3* missense inherited damaging variants were 8%, 34% and 17%, respectively. Further studies with larger cohorts of patients stratified by IQ are therefore needed to achieve appropriate statistical power. Interestingly, in-frame deletions predicted to remove several amino acids in the SHANK2 and SHANK3 proteins were only detected in patients with ASD and their parents, never in controls. Previous functional studies have shown that inherited variants are associated with a statistically significant reduction in the density of synapses, although not as severe as the reduction caused by the *de novo* or truncating mutations [6], [18], [42]–[44]. Together, these genetic and functional data suggest that, although present in healthy parents, some inherited *SHANK* mutations might contribute to the development of ASD.

To date, only non-synonymous mutations in the known exons of the *SHANK* genes had been reported. However, all SHANK genes display several splicing isoforms and possibly some exons were not screened. In addition, other types of mutations such as synonymous mutations or variations in regulatory regions were rarely reported. In our cohort, we did not find synonymous mutations located at alternative splicing sites, but it is warranted that these variations should be reported in future screening.

It has been proposed that abnormal SHANK levels at the synapse might result in the mislocalization, de-clustering and/or functional impairment of several other crucial synaptic proteins such as cytoskeletal regulators and/or neurotransmitter receptors [12] (Table 3).

For *SHANK1* mutations, it is expected that the number of dendrites and glutamatergic synapses will not be dramatically affected (if at all). *SHANK1* mutations might rather lead to an immature neuronal network with a reduced number of large spine heads. Accordingly, male individuals carrying *SHANK1* deletions do not present with language delay or ID and are diagnosed with normal IQ ASD or Asperger syndrome [19]. Interestingly, females who are carrier of a *SHANK1* deletion seem to be protected against ASD suggesting that X-linked genes escaping the X-inactivation process and/or hormonal factors could buffer this type of synaptic alterations.

For *SHANK2* and *SHANK3* mutations, it is expected that affected neurons will have a reduced total number of dendritic spines and synapses. The reduction in mature glutamatergic synapses is expected to affect cognitive functions. Accordingly, most patients with *SHANK2* and *SHANK3* mutations have moderate to severe ID. Individuals with *SHANK3* mutations are usually more severely affected than those carrying *SHANK2* mutations. This difference in severity of cognitive impairment is in agreement with the observation that *SHANK3* mutations are highly penetrant (to our knowledge only one validated *SHANK3* deletion has been reported to be inherited from a mother with moderate ID [45]), while for *SHANK2*, additional genetic/epigenetic factors might be necessary to develop ASD [18], [19], [46].

In summary, our genetic and clinical findings provide additional support for considering *SHANK* mutations in a broad spectrum of patients with ASD. *SHANK* mutations are however not restricted to ASD. *SHANK3* mutations have been identified in patients suffering from schizophrenia and bipolar disorder [45], [47]. More generally, other genes involved in the same synaptic pathway, including neurexin and neuroligin genes, appear to be associated with a variety of neuropsychiatric disorders [11]. Given the broad spectrum of disorders associated with this synaptic pathway, it is important to conduct fine-grained clinical investigations of patients in order to identify the factors that influence the clinical trajectory, clinical manifestations, and outcome of affected individuals [41].

## Materials and Methods

### Study samples for *SHANK* mutations in ASD

Mutation screening of the *SHANK* genes was performed in patients with ASD recruited by the PARIS (Paris Autism Research International Sibpair) study at specialized clinical neuropsychiatric centers located in France and Sweden (Table S2, S3, S4). Ethnicity of the patients and controls was ascertained using genetic data (Figure S6 and Text S1). The autism-spectrum diagnosis was based on the Autism Diagnostic Interview – Revised (ADI-R) [48] and for some of the patients, the Autism Diagnostic Observation Schedule (ADOS) [49]. In Sweden, in few cases, the Diagnostic Interview for Social and Communication Disorders (DISCO-10) [50] was used instead of the ADI-R. IQ was measured with an age-appropriate Weschler scale (WPPSI, Wechsler Preschool and Primary Scale of Intelligence; WISC, Wechsler Intelligence Scale for Children; or WASI, Wechsler Abbreviated Scale of Intelligence). For the most severe and/or non-verbal patients, the Raven's Standard Progressive Matrices were used to measure nonverbal IQ (NVIQ) and the Peabody Picture Vocabulary Test (PPVT-4th edition) to measured receptive vocabulary (RV). In addition, a physical exam was systematically performed to record specifically basic physical parameters (such as height, weight, cranial circumference), dysmorphic features and neurological symptoms. The patients used for the scatter plots of the intellectual quotient and the ADI-R scores, or for the clinical characteristics, or for the prevalence, are indicated in the Table S7.

### Ethics statement

This study was approved by the local Institutional Review Board (IRB) and written informed consents were obtained from all participants of the study. For the patients who were unable to consent for themselves, a parent or legal guardian consented to the study on their behalf. The local IRB are the “Comité de Protection des Personnes” (Île-de-France Hôpital Pitié-Salpêtrière Paris, France); the “Comité de Protection des Personnes Sud Méditerrannée III” (centre hospitalier universitaire de Nîmes, France); the Sahlgrenska Academy Ethics committee (University of Gothenburg, Sweden); SickKids Research Ethics Board (Toronto, Ontario, Canada); Hamilton Integrated Research Ethic Board (HIREB) (Hamilton, Ontario, Canada) and Health Research Ethics Authority (HREA) (St. John's, Newfoundland, Canada).

### *SHANK* copy-number and coding-sequence variants in ASD

*SHANK* copy-number variants were detected with the Illumina Human 1M-Duo BeadChip, and validated by quantitative PCR as previously described [8], [18]. For *SHANK1* and *SHANK2*, the sequencing protocol was adapted from Leblond *et al.* (2012) [18] and Sato *et al.* (2012) [19] (Table S8). For *SHANK3*, because of its high GC content and the genomic sequence errors, several clinical and research centers could not screen exon 11 for mutations [13], [16]. We have now corrected the genomic sequence (Figure S2 and Text S1) and provided a new set of primers that successfully amplified and sequenced all *SHANK3* exons. All *SHANK* primers and sequencing protocols are provided in Table S8.

To ascertain the frequency of *SHANK* mutations in ASD, we included all studies published before April 2012 reporting whole genome copy-number variant screening or *SHANK* mutation screening. We scanned the PubMed database (http://www.ncbi.nlm.nih.gov/pubmed) with combinations of the following keywords: “Autis*”, “mutation*” “Shank*”, Prosap*, “copy number variants”. For *SHANK* copy-number variants, a total of 5 657 cases and 19 163 controls were included, representing 11 studies including this one (Table S3) [8], [9], [13], [14], [18], [19], [34], [35], [51]–[53]. In order to avoid biasing the estimation of the frequency of the copy-number variants, we only included studies that analyzed large numbers of individuals (>200 individuals) and used similar mutation screening procedures (for details see Table S3 and Text S1). To ensure that the cohorts were constituted of independent and unrelated cases and controls, we contacted the corresponding authors of each study. For example, the cohorts used in the following studies: Pinto *et al.* (2010), Moessner *et al.* (2007), Marshall *et al.* (2008), Berkel *et al.* (2010), and Sato *et al.* (2012) contained overlapping samples. The number of non-overlapping samples from these five studies was 1 866 patients with ASD and 15 122 controls, and the total number of non-overlapping samples used in the meta-analysis was 5 657 patients with ASD and 19 163 controls. For patients with ASD, only copy-number variants validated by an independent method were included in the analysis. For controls, the two *SHANK3* deletions reported by Glessner *et al.* (2009) among 2 519 individuals, were not validated and thus should be regarded with caution.

For *SHANK* coding-sequence variants, 10 studies including this one were used (Table S4) [13], [14], [16]–[19], [35], [47]. For all variants, the hg19 coordinates are given. Because of the very low coverage of whole exome sequencing (Figure S3 and Text S1), we excluded mutation screening performed using this approach. Taken together, a total of 760–2 147 patients and 492–1 090 controls were included in the analysis (*SHANK1*: 760 patients and 492 controls; *SHANK2*: 851 patients and 1 090 controls; *SHANK3*: 2 147 patients and 1 031 controls).

### Statistics

The significance of observed differences in copy-number and coding-sequence variants was determined by a two-sided Fisher's exact test on a two-by-two contingency table. We used Bonferroni correction for the multiple testing correction (n_test_ = 12, significant threshold corrected α-value = 0.05/12 = 0.0042). We used G*Power (v3.1, http://www.psycho.uniduesseldorf.de/abteilungen/aap/gpower3) to compute for each test the achieved statistical power (for a two-sided Fisher's exact test) and the power to detect an odd ratio from 1.5 to 3 (two-sided) (Table S12). Prevalence and confidence intervals of single studies were evaluated using Clopper and Pearson method [54]. Heterogeneity between studies was assessed by the Q, I^2^ and Tau^2^ statistics. The Q statistic is a chi-square test for heterogeneity, and the I^2^ and Tau^2^ are the proportion of observed variance in effect sizes across studies for fixed effect model and random effect model, respectively [55]. Zero total event studies (no events in both ASD and controls) were included [56]. The meta-analysis was performed using the classical inverse variance for both fixed effects model and random effects model. To avoid population stratification bias in the calculation of the odds ratio (OR), studies without a control group were excluded (i.e. Boccuto et al. 2012 and Bremer et al. 2012) (Figure 2, 3, and Table 1). If any contingency tables contained zero values, a continuity correction was applied to the relevant tables [57]. For all the calculations and illustrations the R statistical software, and “metafor” and “meta” packages were used.

## Supporting Information

Figure S1 Genomic structure and phylogeny of *SHANK* family. **A.** Genomic structure of human *SHANK* genes. Conserved domains of protein interaction are represented in color: ANK (red), SH3 (orange), PDZ (blue) and SAM (green). Black stars identify the alternative spliced exons and turquoise stars the alternative spliced exons specifically retained in the human brain. Grey bars indicate CpGs islands and the arrows the different isoforms. The areas of the human genome with missing sequence are indicated by purple rectangles. **B.** Phylogenetic tree of SHANK proteins. SHANK1 was blasted with non-redundant protein sequence database and the tree was produced using the Neighbor joining method with a maximum of sequence difference = 0.85 and the Grishin Distance.
(TIF)

Figure S2 Genome errors covering *SHANK3*. **A.** Representation of the reference sequence and mRNA of *SHANK3* in hg19 (http://genome.ucsc.edu/). Before the update of hg19 in March 2012, *SHANK3* was supported by *NM_001080420.1* carrying annotation and sequence errors. The false exon 11 was corrected in March 2012, but the real exon 11 still contained a wrong sequence with a gap. Using a combination of PCR and BLAST experiments, we corrected this sequence gb_JX122810. **B.** The clustalW2 alignment (http://www.ebi.ac.uk/Tools/msa/clustalw2/) shows the gap still present in hg19 and located in the 5′UTR and coding region of the exon 11 of *SHANK3*. JX122810 is the GenBank (http://www.ncbi.nlm.nih.gov/genbank/) accession number of the validated intron flanking and exon 11 sequences of *NM_033517*. E11, Exon 11; E12, Exon 12; gb, GenBank; hg, human genome; EST, Expressed Sequence Tag.
(TIF)

Figure S3 Read depth for *SHANK* genes using whole genome or exome sequencing. The average read depth of whole genome sequence from Complete Genomics (n = 54) and whole exome sequence from NHLBI GO Exome Sequencing Project – Exome Variant server (n = 3 510) are indicated in black and gray, respectively. The Y axis shows the average read number per nucleotide. On the X axis, the nucleotide positions are according to *NM_016148* (*SHANK1*), *NM_012309* (*SHANK2*), and *NM_033517* (*SHANK3*) from NCBI37/hg19. The percentage of GC is calculated from sequences with size equal to 5 nucleotides. The arrows show the direction of the transcription. Truncating, deleterious and neutral coding-sequence variants are indicated in red, orange and green, respectively. Coding-sequence variants identified in ASD only, or in controls only, or in both ASD and controls are indicated by a star, a square or a circle, respectively. Avg, Average; ME, Multi-Ethnic; EA, European American; EVS, Exome Variant Server; CG, Complete Genomics.
(TIF)

Figure S4 Characterization of the *de novo* deletions of *SHANK2* and *SHANK3* identified in this study. **A.** A *de novo* deletion of 1.8 Mb including *SHANK2* was identified in a patient with ASD (RDB_30769) using the HumanCytoSNP-12 Illumina array. FISH studies using the “RP11-102B19” probe covering *SHANK2* showed one normal chromosome 11 with one green spot on 11q13, and the second chromosome 11 without the green signal. The parent's metaphase karyotype shows a green spot on both chromosomes 11. White arrows indicate the localization of the *SHANK2* probe on chromosome 11. **B.** A *de novo* deletion of 1.5 Mb was identified in a patient with ASD (AU029) using the Illumina Human 1M-Duo SNP array. The results of the SNP arrays are represented using SnipPeep (CNV viewer; http://snippeep.sourceforge.net/). Each dot shows Log R Ratio (LRR; in red) and B allele frequency (BAF; in green). QuantiSNP (CNV calling algorithm; CN = Copy Number) score is represented with a blue line and indicates the deletion size. **C.** Two *de novo* deletions altering *SHANK3* were identified in two independent patients (AUN_006 & AUN_007) with ASD and ID using Multiplex Ligation-dependent Probe Amplification (MLPA) (probemix P188-B2, P343-C1& P339-A1 - MRC-Holland). The first patient AUN_006 carried a deletion including *SHANK3* (exons 9 to 22), *ACR* and *RABL2B* with a breakpoint in intron 8 of *SHANK3*. The second patient AUN_007 carried a deletion of *SHANK3* (exons 22 only) and *ACR* (exons 1 to 3). The parents of AUN_006 and AUN_007 probands were negative for *SHANK3* CNV (FISH and MLPA analysis not shown). ASD, Autism Spectrum Disorder; ID, Intellectual Disability; FISH, Fluorescent In Situ Hybridization.
(TIF)

Figure S5 Pedigrees of the families carrying *de novo*/truncating *SHANK3* mutations. The chromatograms obtained after Sanger sequencing show eight new truncating mutations altering *SHANK3* detected in patients with ASD. When the DNA of the both parents was available (7 out of 9 families), all the mutations were found to be *de novo*. The arrows indicate the frame-shift. The patient carrying the Q1243X was found during our screening of exon 21 in 138 individuals with ASD.
(TIF)

Figure S6 MDS-Plot: Genetic ancestry of patients with ASD and controls. The multidimensional scaling (MDS) plot pictures the genetic distance between individuals. The density of the genetic ancestry of the HapMap populations (European, Asian, African, Mexican and Indian) allows confirming the European ancestry of the majority of the individuals from PARIS and SUVIMAX cohorts (n = 430 ASD and n = 837 controls). Patients and controls with no *SHANK* mutation are represented by blue crosses and blue circles, respectively. Patients and controls with *SHANK* mutations are indicated in red and in green, respectively. *SHANK3* mutations are identified by diamonds, *SHANK2* by triangles and *SHANK1* by squares. Diamonds, triangles or squares are empty when the mutation is a missense and full when the mutation is truncating (CVS or CNV). CVS, Coding-sequence variant; CNV, Copy-number variant.
(TIF)

Table S1 Genomic sequence covering exons 8 and 9 of human *SHANK2* and exon 11 of human *SHANK3*. The exonic and intronic sequences are indicated in blue upper case and in black lower case, respectively. The primers used for the amplification of each exon are indicated by the black boxes. The alternative stop in exon 21b of SHANK3 is underlined.
(DOC)

Table S2 Description of the cohort PARIS used for the screening of *SHANK* copy-number variants and coding-sequence variants. PARIS, Paris Autism Research International Sibpair; IQ, Intelligence Quotient.
(DOC)

Table S3 Description of the cohorts used for the analysis of *SHANK* copy-number variants. ^a,b^ Indicate the publications with overlap in the cohorts. * The total number of independent cases or controls is not the addition of the cohorts from each study due to the redundancy of the cases and controls tested from these publications. The independent numbers were obtained in collaboration with the authors of the corresponding publications (see Material & Method in supplementary Appendix). The parental DNA of controls was not available. PARIS, Paris Autism Research International Sibpair study; SSC, Simons Simplex Collection; AGP, Autism Genome Project; ACC, Autism Case Control; SAGE, Study of Addiction: Genetics and Environment; CHOP, Children's Hospital of Philadelphia; WTCCC2, Wellcome Trust Case Control Consortium; AGRE, Autism Genetic Resource exchange; NIMH, National Institute of Mental Health; SNP, Single Nucleotide Polymorphism; CEU, Utah residents with Northern and Western European ancestry from the CEPH collection; BAC, Bacterial Artificial Chromosome; CGH, Comparative Genomic Hybridization; ROMA, Representational Oligonucleotide Microarray Analysis; ADI-R, Autism Diagnostic Interview-Revised; ADOS, Autism Diagnostic Observation Schedule; DSM-IV-TR, Diagnostic and Statistical Manual of Mental Disorders, Fourth Edition-Text Revision; DISCO, Diagnostic Interview for Social and Communication Disorders; IQ, Intellectual Quotient; RPM, Raven's Progressive Matrices; PPVT, Peabody Picture Vocabulary Test.
(DOC)

Table S4 Description of the cohorts used for the analysis of *SHANK* coding-sequence variants. ^a^ In this study, controls were tested using allelic discrimination by TaqMan technology and only for the variations identified in ASD. The parental DNA of controls was not available. SUVIMAX, Supplémentation en Vitamines et Minéraux Antioxydants; ADI-R, Autism Diagnostic Interview-Revised; ADOS, Autism Diagnostic Observation Schedule; DSM-IV-TR, Diagnostic and Statistical Manual of Mental Disorders, Fourth Edition-Text Revision; DISCO, Diagnostic Interview for Social and Communication Disorders; IQ, Intellectual Quotient; RPM, Raven's Progressive Matrices; PPVT, Peabody Picture Vocabulary Test; SSC, Simons Simplex Collection.
(DOC)

Table S5 Prevalence of *SHANK* CNVs and coding-sequence variants in patients with ASD and controls. ^a^ Sato et al. (2012), Durand et al. (2007) Leblond et al. (2012) contained overlapping cohorts. For this study, the French and Swedish cases are in Leblond et al (2012) and the Canadian cases are in Sato et al. (2012). ^b^The controls from Sato *et al.* 2012 are not included here because they were only tested by Taqman for the variants identified in ASD. ^c^The two *SHANK3* deletions reported by Glessner et al. (2009) in control subjects have not been validated and should be interpreted with caution. CSV, Coding-Sequence Variant; 95CI, 95% Confidence Interval.
(DOC)

Table S6 Prevalence of *SHANK* CNVs and coding-sequence variants in ASD patients with or without ID. ^a^ Sato et al. (2012) and Leblond et al. (2012) contained overlapping cohorts. For this study, the French and Swedish cases are in Leblond et al (2012) and the Canadian cases are in Sato et al. (2012). ^b^ The controls from Sato *et al.* 2012 are not included here because they were only tested by Taqman for the variants identified in ASD. CSV, Coding-Sequence Variant.
(DOC)

Table S7 Genetic and clinical features of ASD patients carrying *de novo*/deleterious *SHANK* mutations. **^a^** Mother showed anxiety and shyness. **^b^** Mutations not present in the mother and father not tested (DNA unavailable). **^c^** Father had a balanced translocation t(14;22)(p11.2;q13.33). **^d^** Mother had a balanced translocation t(14;22)(p11.2;q13.33). **^e^** No epilepsy, but abnormal EEG with bilateral epileptiform discharges. **^f^** Relatives of proband IV-1. **^g^** Case reports not reported in a systematic screening of *SHANK2* or whole genome analysis. **^h^** Patient detected in an additional mutation screening of *SHANK3* exon 21. **^i^** Relatives of AU016_3. **^j^** Not included in the figure: large deletion involving numerous genes or complex chromosomal rearrangement. **^k^** For *SHANK3*, we only included in figure 2 the patients from the PARIS cohort. **^l^** No pictures available. **^m^** The phenotypic features were characteristic to the Phelan-Mcdermid syndrome. AS, absence seizures; ASD, autism spectrum disorder; Asp, Asperger syndrome; Aut, autism; CNV, copy number variant, CSV, coding-sequence variants; del, deletion; f, female; ID; inh, inherited; m, male; U, unknown; y, years; GTCS, generalized tonic-clonic seizures.
(DOC)

Table S8 Primers used for mutation screening of *SHANK1* and *SHANK3*. The red sequences correspond to the M13 adaptor (M13F = TGTAAAACGACGGCCAGT & M13R = GGATAACAATTTCACACAGG). PCR, Polymerase Chain Reaction; TpQ, Tampon Q (from Qiagen); DMSO, Dimethyl sulfoxide.
(DOC)

Table S9 *SHANK1* coding-sequence variants identified in 760 patients with ASD and 492 controls. ^a^Nucleotide positions are according to *NM_016148* from NCBI37/hg19 on the positive DNA strand (chromosome 19). The patients with ASD used for this analysis came from this study (n = 240) and from the study of Sato *et al.* (2012) (n = 509). The Grantham matrix and GERP scores were obtained from SeattleSeq Annotation 134. We used the Fisher's exact test (2-sided) and Pearson's Chi-squared test with Yates' continuity correction. P, p-value; ASD, Autism Spectrum Disorder; MAF, Minor Allele Frequency; GERP, Genomic Evolutionary Rate Profiling; pph2_class, polyphen-2_class.
(DOC)

Table S10 *SHANK2* coding-sequence variants identified in 851 patients with ASD and 1 090 controls. ^#^Indicates *de novo* mutations. ^a^Nucleotide positions are according to *NM_012309.3* from NCBI37/hg19 on the positive DNA strand (chromosome 11). ^b^Maximum Grantham score (215) given for non-sense variants. The patients with ASD and the controls used for this analysis came from Leblond *et al.* (2012) (455 ASD & 431 controls) and from the study of Berkel *et al.* (2010) (396 ASD & 659 controls). The Grantham matrix and GERP scores were obtained from SeattleSeq Annotation 134. We used the Fisher's exact test (2-sided) and Pearson's Chi-squared test with Yates' continuity correction. P, p-value; ASD, Autism Spectrum Disorder; MAF, Minor Allele Frequency; GERP, Genomic Evolutionary Rate Profiling; pph2_class, polyphen-2_class.
(DOC)

Table S11 *SHANK3* coding-sequence variants identified in 2 147 patients with ASD and 1 031 controls. ^#^Indicates *de novo* mutations. ^+^Q1243X was identified during an additional screen (nASD = 138) of exon 21 of SHANK3 and was not included in the meta-analysis. ^a^Nucleotide positions are according to *NM_033517* from NCBI37/hg19 on the positive DNA strand (chromosome 22); ^b^For the variants with MAF>1%, the frequency was assessed only in PARIS cohort. ^c^Average GERP score for two sites flanking the insertion or average GERP score for deleted nucleotides; ^d^Maximum Grantham score (215) given for splice, non-sense and frameshifting variants. The patients with ASD and the controls used for this analysis came from this study (n = 429) and from the studies reported in Table S5. The Grantham matrix and GERP scores were obtained from SeattleSeq Annotation 134. We used the Fisher's exact test (2-sided) and Pearson's Chi-squared test with Yates' continuity correction. P, p-value; ASD, Autism Spectrum Disorder; MAF, Minor Allele Frequency; GERP, Genomic Evolutionary Rate Profiling, pph2_class, polyphen-2_class.
(DOC)

Table S12 Statistical power of the association between *SHANK* damaging missense variants and ASD. *Two-sided Fisher's exact test.
(DOC)

Text S1 Supplementary method describing the genomic structure of the *SHANK* genes, coverage of *SHANK* genes by exome sequencing, cytogenetic analysis and FISH, multiplex ligation-dependent probe amplification (MLPA), genetic ancestry of patients with ASD and controls.
(DOC)
