# Supplementary figures and images for: Meta-analysis of SHANK Mutations in Autism Spectrum Disorders: A Gradient of Severity in Cognitive Impairments
Source: PLoS Genet. 2014 Sep 4;10(9):e1004580. doi: 10.1371/journal.pgen.1004580 (PMC4154644; doi:10.1371/journal.pgen.1004580)

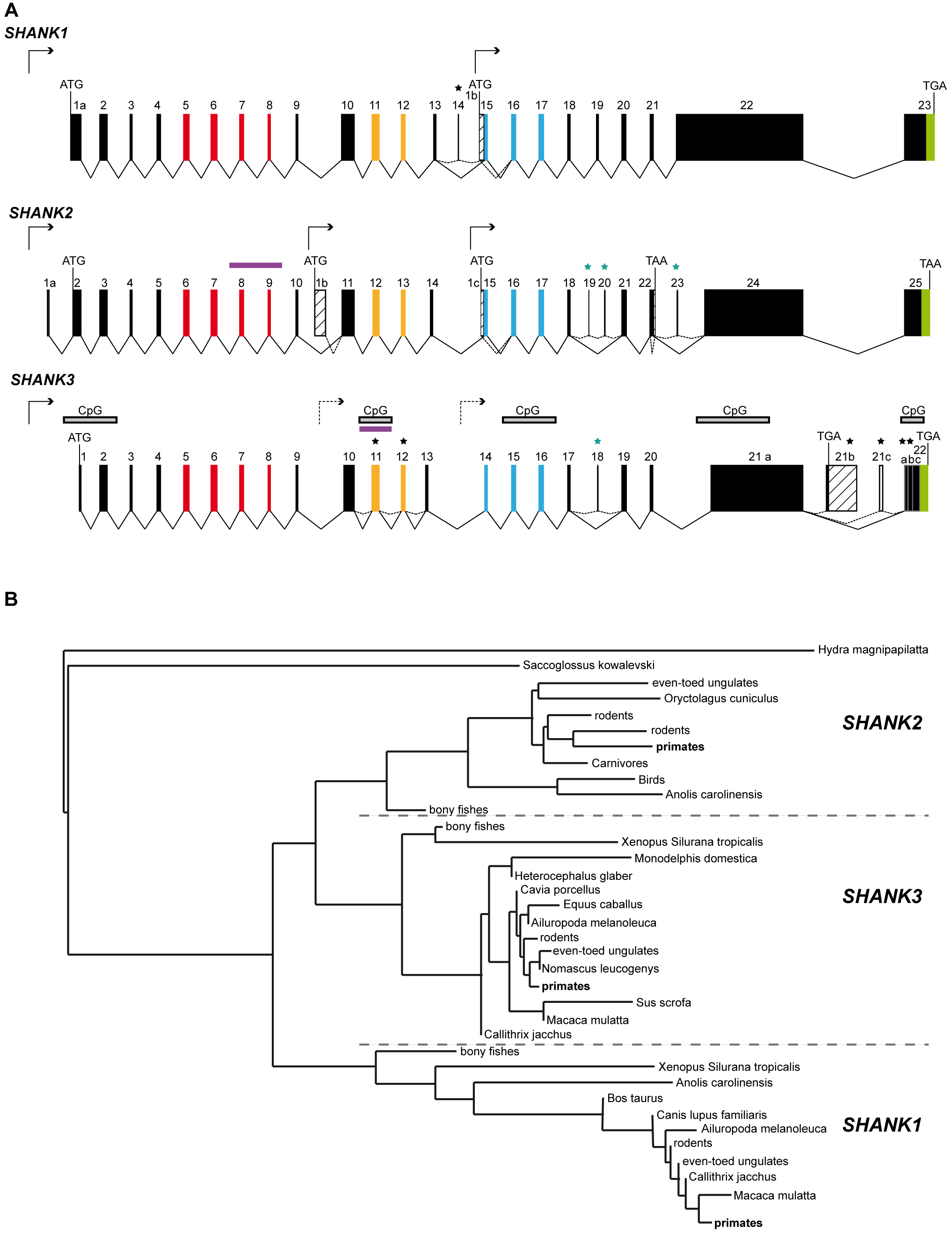

Supplement: Figure S1 — Genomic structure and phylogeny of SHANK family. A. Genomic structure of human SHANK genes. Conserved domains of protein interaction are represented in color: ANK (red), SH3 (orange), PDZ (blue) and SAM (green). Black stars identify the alternative spliced exons and turquoise stars the alternative spliced exons specifically retained in the human brain. Grey bars indicate CpGs islands and the arrows the different isoforms. The areas of the human genome with missing sequence are indicated by purple rectangles. B. Phylogenetic tree of SHANK proteins. SHANK1 was blasted with non-redundant protein sequence database and the tree was produced using the Neighbor joining method with a maximum of sequence difference = 0.85 and the Grishin Distance. (TIF) [file pgen.1004580.s001.tif]

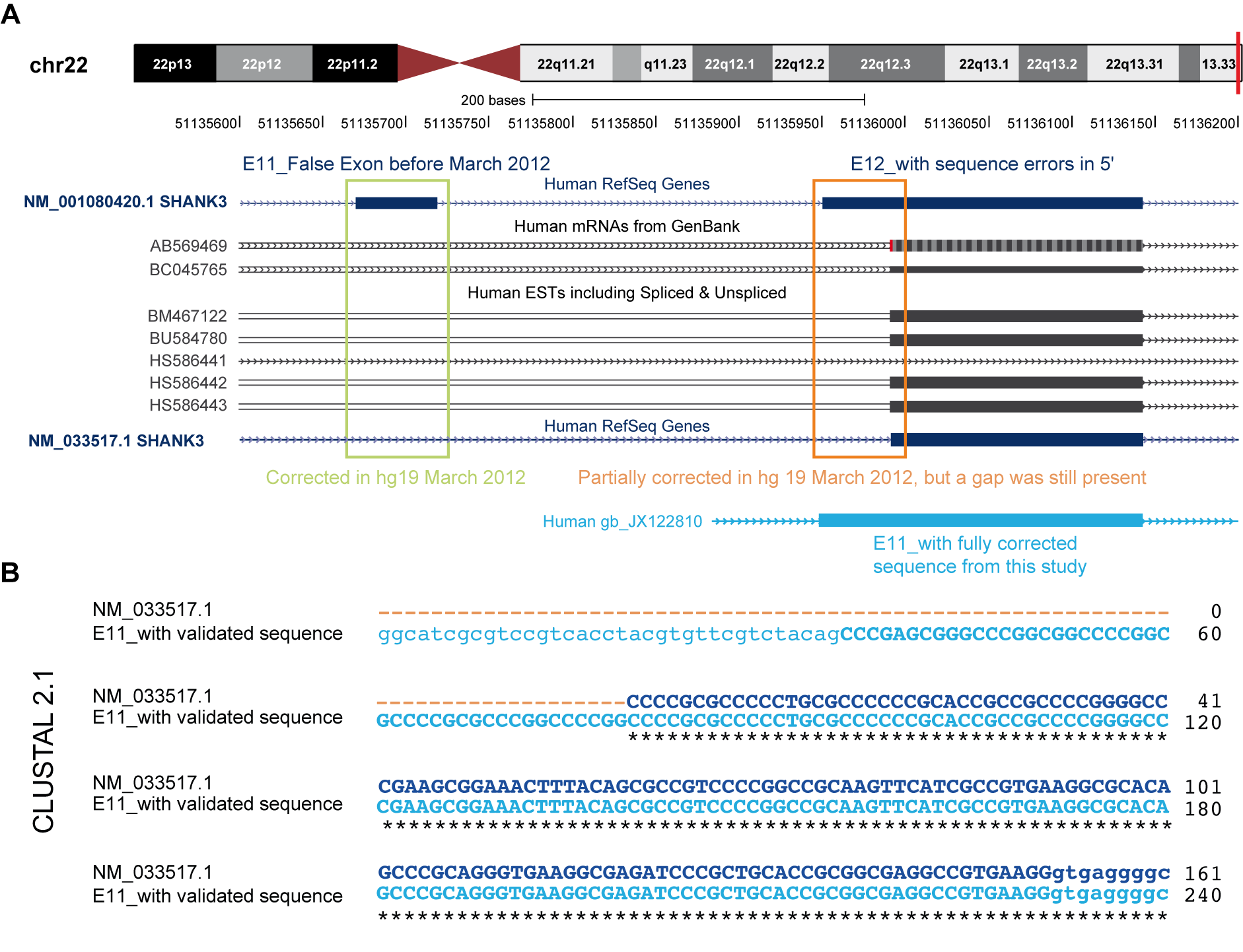

Supplement: Figure S2 — Genome errors covering SHANK3. A. Representation of the reference sequence and mRNA of SHANK3 in hg19 (http://genome.ucsc.edu/). Before the update of hg19 in March 2012, SHANK3 was supported by NM_001080420.1 carrying annotation and sequence errors. The false exon 11 was corrected in March 2012, but the real exon 11 still contained a wrong sequence with a gap. Using a combination of PCR and BLAST experiments, we corrected this sequence gb_JX122810. B. The clustalW2 alignment (http://www.ebi.ac.uk/Tools/msa/clustalw2/) shows the gap still present in hg19 and located in the 5′UTR and coding region of the exon 11 of SHANK3. JX122810 is the GenBank (http://www.ncbi.nlm.nih.gov/genbank/) accession number of the validated intron flanking and exon 11 sequences of NM_033517. E11, Exon 11; E12, Exon 12; gb, GenBank; hg, human genome; EST, Expressed Sequence Tag. (TIF) [file pgen.1004580.s002.tif]

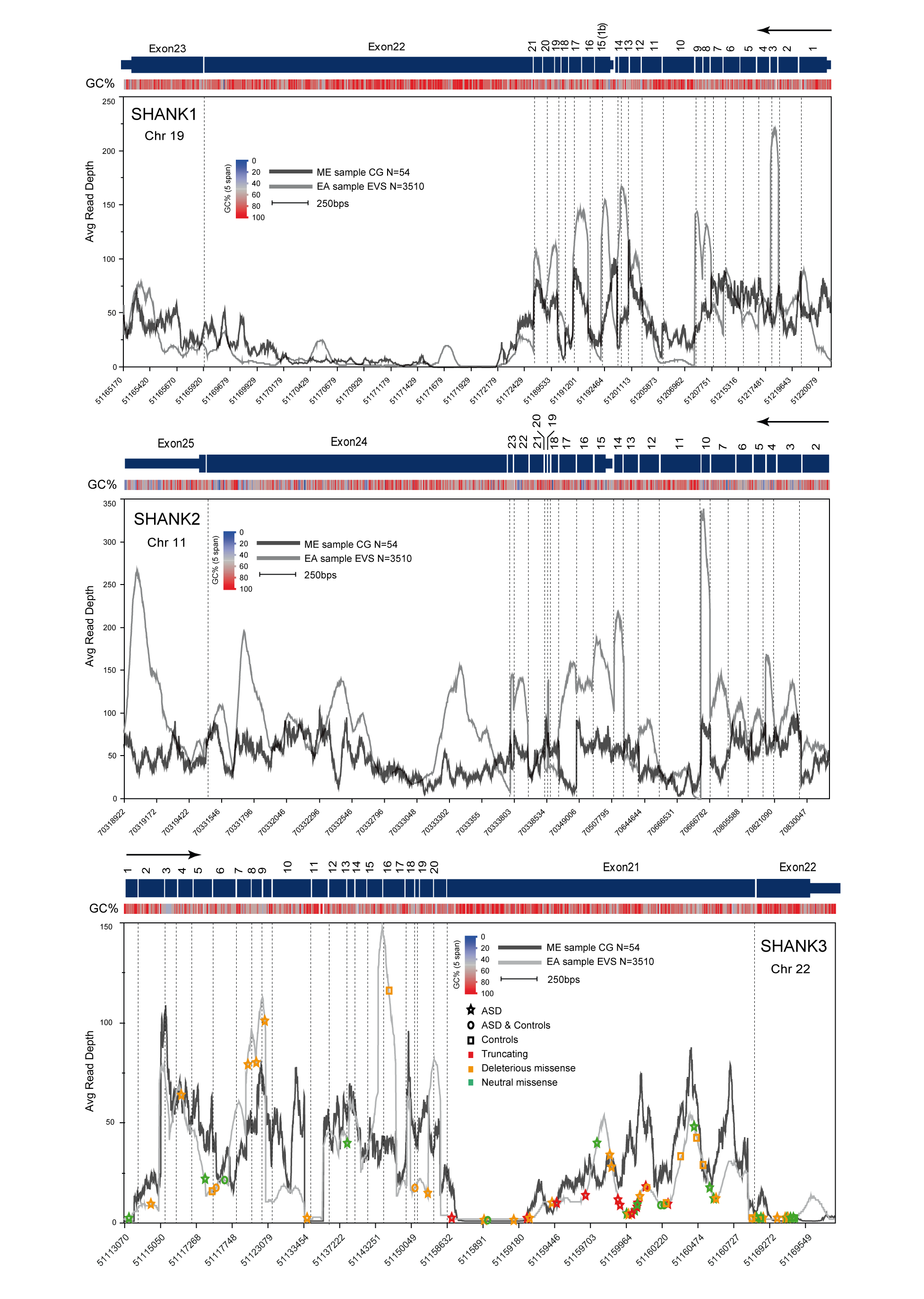

Supplement: Figure S3 — Read depth for SHANK genes using whole genome or exome sequencing. The average read depth of whole genome sequence from Complete Genomics (n = 54) and whole exome sequence from NHLBI GO Exome Sequencing Project – Exome Variant server (n = 3 510) are indicated in black and gray, respectively. The Y axis shows the average read number per nucleotide. On the X axis, the nucleotide positions are according to NM_016148 (SHANK1), NM_012309 (SHANK2), and NM_033517 (SHANK3) from NCBI37/hg19. The percentage of GC is calculated from sequences with size equal to 5 nucleotides. The arrows show the direction of the transcription. Truncating, deleterious and neutral coding-sequence variants are indicated in red, orange and green, respectively. Coding-sequence variants identified in ASD only, or in controls only, or in both ASD and controls are indicated by a star, a square or a circle, respectively. Avg, Average; ME, Multi-Ethnic; EA, European American; EVS, Exome Variant Server; CG, Complete Genomics. (TIF) [file pgen.1004580.s003.tif]

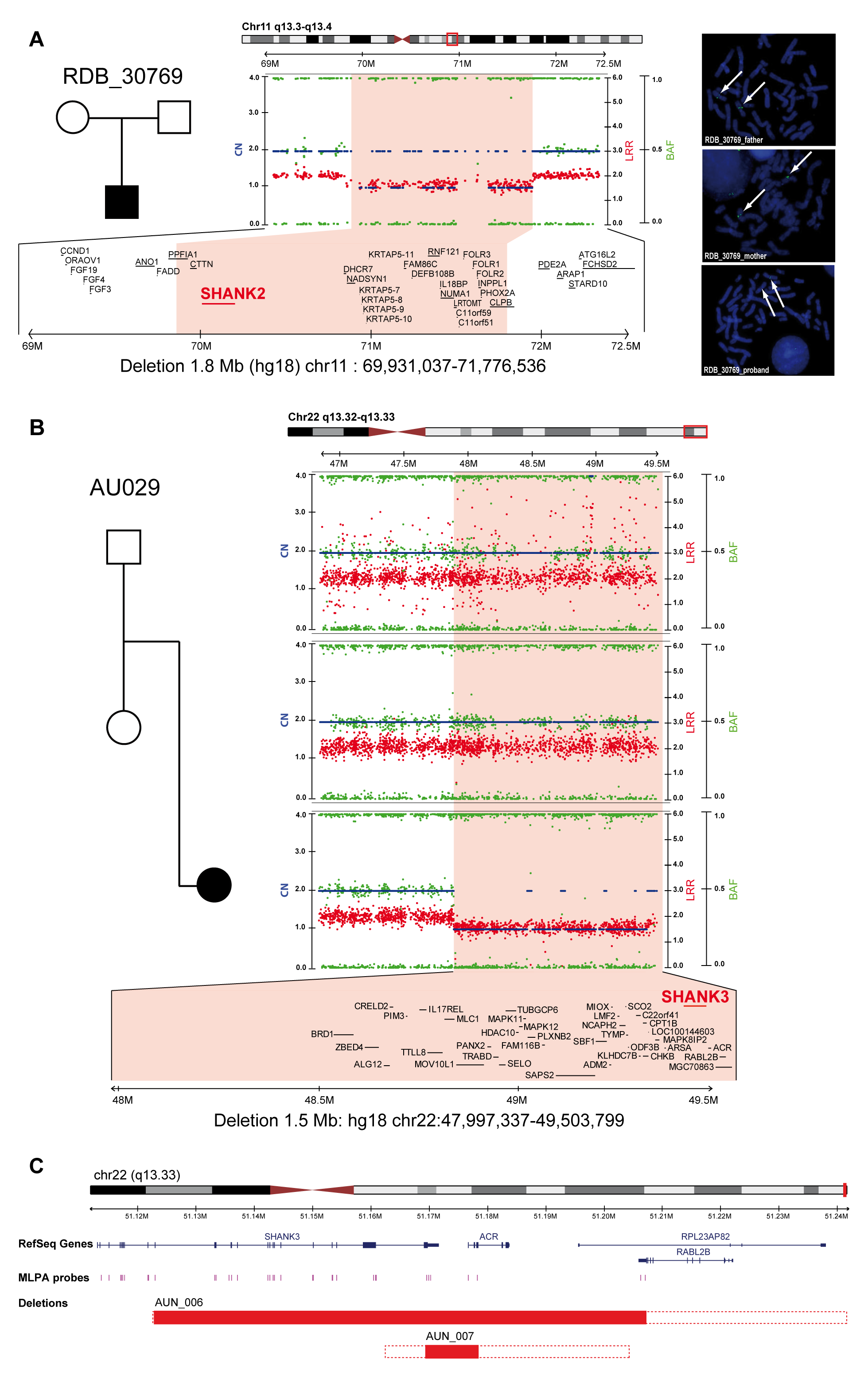

Supplement: Figure S4 — Characterization of the de novo deletions of SHANK2 and SHANK3 identified in this study. A. A de novo deletion of 1.8 Mb including SHANK2 was identified in a patient with ASD (RDB_30769) using the HumanCytoSNP-12 Illumina array. FISH studies using the “RP11-102B19” probe covering SHANK2 showed one normal chromosome 11 with one green spot on 11q13, and the second chromosome 11 without the green signal. The parent's metaphase karyotype shows a green spot on both chromosomes 11. White arrows indicate the localization of the SHANK2 probe on chromosome 11. B. A de novo deletion of 1.5 Mb was identified in a patient with ASD (AU029) using the Illumina Human 1M-Duo SNP array. The results of the SNP arrays are represented using SnipPeep (CNV viewer; http://snippeep.sourceforge.net/). Each dot shows Log R Ratio (LRR; in red) and B allele frequency (BAF; in green). QuantiSNP (CNV calling algorithm; CN = Copy Number) score is represented with a blue line and indicates the deletion size. C. Two de novo deletions altering SHANK3 were identified in two independent patients (AUN_006 & AUN_007) with ASD and ID using Multiplex Ligation-dependent Probe Amplification (MLPA) (probemix P188-B2, P343-C1& P339-A1 - MRC-Holland). The first patient AUN_006 carried a deletion including SHANK3 (exons 9 to 22), ACR and RABL2B with a breakpoint in intron 8 of SHANK3. The second patient AUN_007 carried a deletion of SHANK3 (exons 22 only) and ACR (exons 1 to 3). The parents of AUN_006 and AUN_007 probands were negative for SHANK3 CNV (FISH and MLPA analysis not shown). ASD, Autism Spectrum Disorder; ID, Intellectual Disability; FISH, Fluorescent In Situ Hybridization. (TIF) [file pgen.1004580.s004.tif]

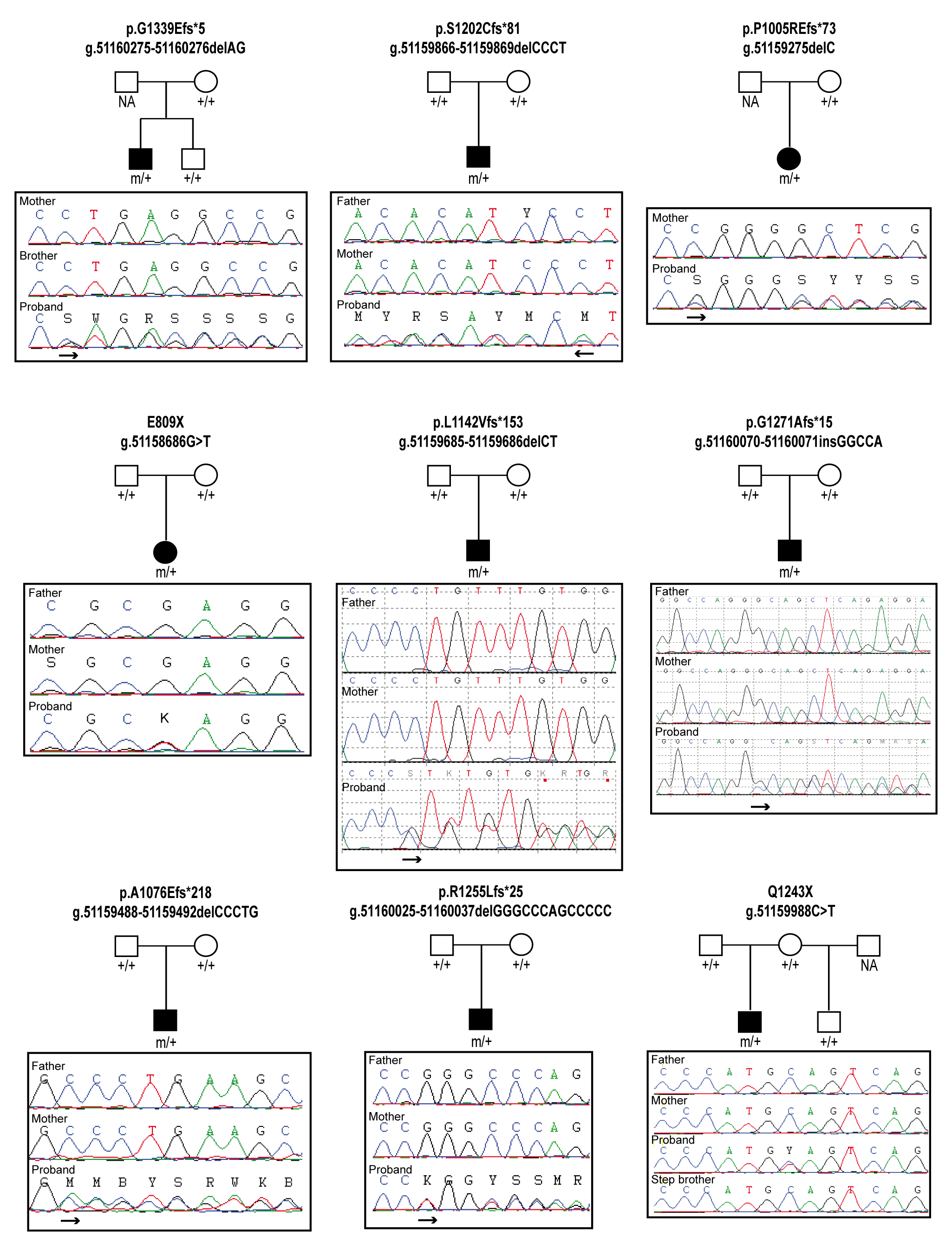

Supplement: Figure S5 — Pedigrees of the families carrying de novo/truncating SHANK3 mutations. The chromatograms obtained after Sanger sequencing show eight new truncating mutations altering SHANK3 detected in patients with ASD. When the DNA of the both parents was available (7 out of 9 families), all the mutations were found to be de novo. The arrows indicate the frame-shift. The patient carrying the Q1243X was found during our screening of exon 21 in 138 individuals with ASD. (TIF) [file pgen.1004580.s005.tif]

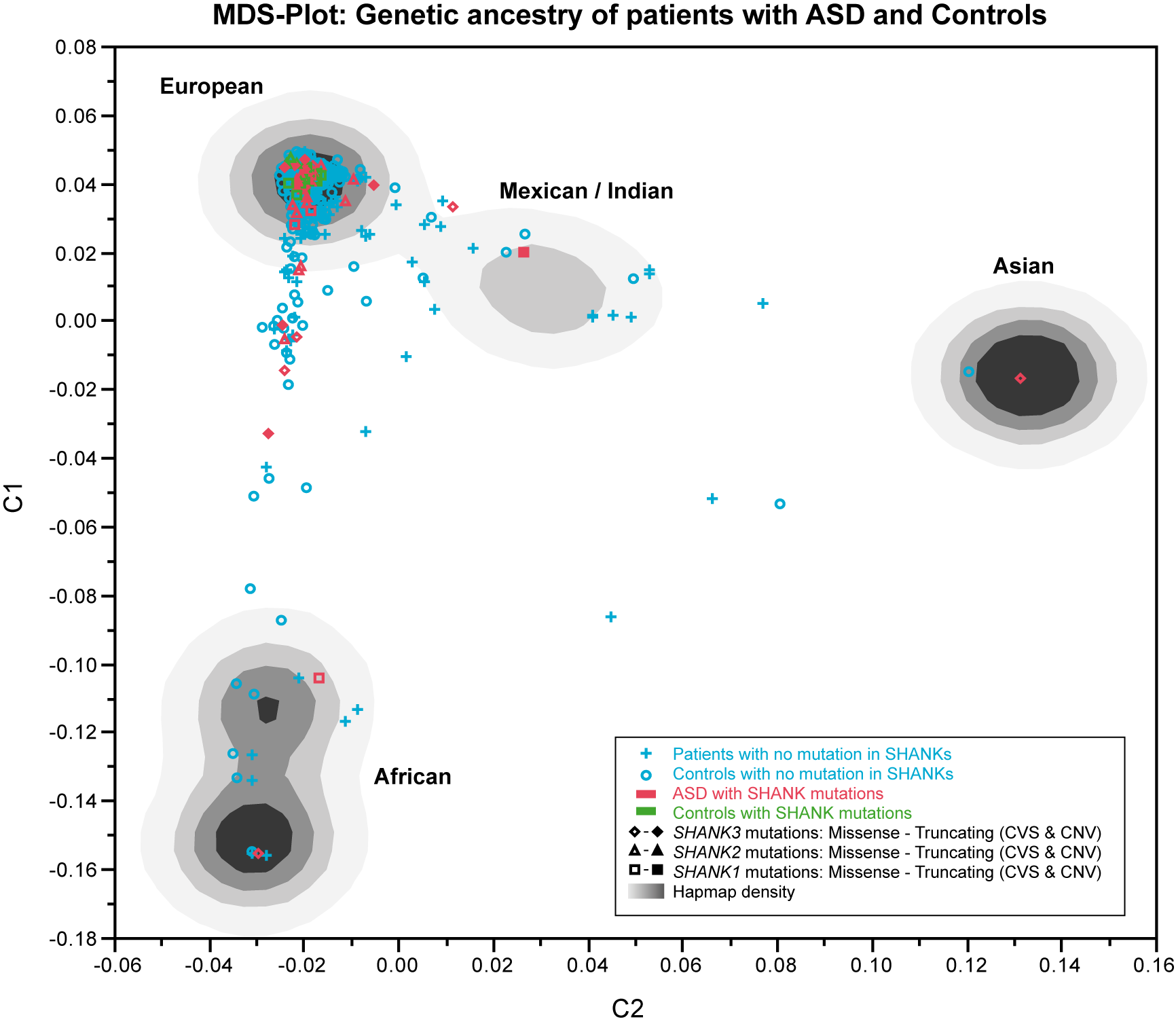

Supplement: Figure S6 — MDS-Plot: Genetic ancestry of patients with ASD and controls. The multidimensional scaling (MDS) plot pictures the genetic distance between individuals. The density of the genetic ancestry of the HapMap populations (European, Asian, African, Mexican and Indian) allows confirming the European ancestry of the majority of the individuals from PARIS and SUVIMAX cohorts (n = 430 ASD and n = 837 controls). Patients and controls with no SHANK mutation are represented by blue crosses and blue circles, respectively. Patients and controls with SHANK mutations are indicated in red and in green, respectively. SHANK3 mutations are identified by diamonds, SHANK2 by triangles and SHANK1 by squares. Diamonds, triangles or squares are empty when the mutation is a missense and full when the mutation is truncating (CVS or CNV). CVS, Coding-sequence variant; CNV, Copy-number variant. (TIF) [file pgen.1004580.s006.tif]
