# Supplementary material for: Meta-analysis of SHANK Mutations in Autism Spectrum Disorders: A Gradient of Severity in Cognitive Impairments
Source: PLoS Genet. 2014 Sep 4;10(9):e1004580. doi: 10.1371/journal.pgen.1004580 (PMC4154644; doi:10.1371/journal.pgen.1004580)
Supplement: Table S1 — Genomic sequence covering exons 8 and 9 of human SHANK2 and exon 11 of human SHANK3. The exonic and intronic sequences are indicated in blue upper case and in black lower case, respectively. The primers used for the amplification of each exon are indicated by the black boxes. The alternative stop in exon 21b of SHANK3 is underlined. (DOC) [file pgen.1004580.s007.doc]

Table S1: Genomic sequence covering exons 8 and 9 of human *SHANK2* and exon 11 of human *SHANK3*

| Gene | Exon | size | Sequence |
| --- | --- | --- | --- |
| *SHANK2 (*JX122808 ) | 8 | 117 pb | ccgagtggctggtgtactttgtgtgtgcatgcggctgtcgtggcggagggttcctgattcacagcctgggttctgtgtttcag**gcctgcaggtacgggcacgtgcagcacctggagcacctgctgttctacggggcagacatgagtgcccagaatgcctcggggaacacggccttgcacatctgcgccctctacaaccag**gtgagtgctgagcgcatttaaaggaaataatgtatacagtggtccatttccaaaacaaagtgccttaaattggcttacgtcagc |
| *SHANK2* (JX122809) | 9 | 78 pb | gcctcacctgacagcatacaggagccagatgaaagcaacatgctttcccttttaactgtgcacttctgtttccctggtttcccgtgagcttgagactctggacagatttctttggagaatgtctgtgctaccattttacaaacaggtttctccttttctatttgcag**gacagctgtgcaagagtgcttctgtttcgaggcggaaataaggagttaaaaaactacaacagccagactccatttcag**gtaaaagagatctcagcatcaggaaggatgtgtgtggatcacagtgggtgatggctctgcgtctccacggtcgtgtttgta |
| *SHANK3* (JX122810) | 11 | 194 pb | ggcatcgcgtccgtcacctacgtgttcgtctacag**cccgagcgggcccggcggccccggccccgcgcccggccccggccccgcgccccctgcgccccccgcaccgccgccccggggcccgaagcggaaactttacagcgccgtccccggccgcaagttcatcgccgtgaaggcgcacagcccgcagggtgaaggcgagatcccgctgcaccgcggcgaggccgtgaagG**gtgaggggcgcgggggggcgcgggggggcgggcccggcgcggggagggggcggcgccgcgcgcggtgctggccgggccggggcagtggctctggggtctcctc |
| *SHANK3* | 21b | 55 pb | ggctccttccaaagaggagcccttcgggcccgtgggctgcatggatgctggcggcagagctggtcatcccccacccgcccccttgtctgcctttttaaagctgcttttgccttctgtgcccctag**GTCTCCCCTCTCCTCTTTGGGTCTGGGGGGGTGGTATGTGGATGCCACCTCTTGA**ctcctgcttcttgctgcctggaagaccaacctagtgggccccgtactgtcagccttggaggacagagttcacagcgtagcaacgtgttcagaacttaaggactttgcaggt |
| *SHANK3* | 21c | 107 pb | ggcagagggcggagtggggagggtgtggcaaggccagacctctctcactggctgagagtcactgggaaacagtgagacggtgcaggattcaaatgtaggtggaaag**acgtggggccgtgggggcctcagtgtgatctggactcagcctcttcagcgtggctgctggaggtgttcgtgggtgacggtgcctggtgaagtatcatgtgttagagg**gtgggtggcccagaagctctctgggaggctggcaggtgctgtgatgtggctgtttggcggccggagcctggccagggaggtggccagacggctgagagtgacagtggg |
| *SHANK3* | 22a | 82 pb | **GCTCTTCAGCAGCCTCGGTGAGCTGAGCTCCATTTCAGCGCAGCGCAGCCCCGGGGGCCCGGGCGGCGGGGCCTCGTACTCG** |
| *SHANK3* | 22b | 144 pb | **GTGAGGCCCAGTGGCCGCTACCCCGTGGCGAGACGCGCCCCGAGCCCGGTGAAGCCCGCGTCGCTGGAGCGGGTGGAGGGGCTGGGGGCGGGCGCGGGGGGCGCAGGGCGGCCCTTCGGCCTCACGCCCCCCACCATCCTCAAG** |
| *SHANK3* | 22c | 366 pb | **TCGTCCAGCCTCTCCATCCCGCACGAGCCCAAGGAGGTGCGCTTCGTGGTGCGCAGCGTGAGCGCGCGCAGTCGCTCCCCCTCGCCGTCGCCGCTGCCCTCGCCCGCGTCCGGCCCCGGCCCCGGCGCCCCCGGCCCACGCCGACCCTTCCAGCAGAAGCCGCTGCAGCTCTGGAGCAAGTTCGACGTGGGCGACTGGCTGGAGAGCATCCACCTAGGCGAGCACCGCGACCGCTTCGAGGACCATGAGATAGAAGGCGCGCACCTACCCGCGCTTACCAAGGACGACTTCGTGGAGCTGGGCGTCACGCGCGTGGGCCACCGCATGAACATCGAGCGCGCGCTCAGGCAGCTGGACGGCAGCTGA** |
