# Supplementary material for: Meta-analysis of SHANK Mutations in Autism Spectrum Disorders: A Gradient of Severity in Cognitive Impairments
Source: PLoS Genet. 2014 Sep 4;10(9):e1004580. doi: 10.1371/journal.pgen.1004580 (PMC4154644; doi:10.1371/journal.pgen.1004580)
Supplement: Table S2 — Description of the cohort PARIS used for the screening of SHANK copy-number variants and coding-sequence variants. PARIS, Paris Autism Research International Sibpair; IQ, Intelligence Quotient. (DOC) [file pgen.1004580.s008.doc]

Table S2: Description of the cohort PARIS used for the screening of *SHANK* copy-number variants and coding-sequence variants

|  |  | **Sex** | | | **Intellectual disability** | | |
| --- | --- | --- | --- | --- | --- | --- | --- |
|  | **Total** | **Male** | **Female** | **Unknown** | **IQ>70** | **IQ<70** | **Unknown** |
| Autism | n=743 | 558 | 146 | 39 | 125 | 453 | 165 |
| Atypical autism | n=49 | 38 | 11 | 0 | 8 | 30 | 11 |
| Asperger syndrome | n=128 | 108 | 20 | 0 | 84 | 6 | 38 |
| **Total** | **n=920** | **704** | **177** | **39** | **217** | **489** | **214** |
