# Supplementary material for: Meta-analysis of SHANK Mutations in Autism Spectrum Disorders: A Gradient of Severity in Cognitive Impairments
Source: PLoS Genet. 2014 Sep 4;10(9):e1004580. doi: 10.1371/journal.pgen.1004580 (PMC4154644; doi:10.1371/journal.pgen.1004580)
Supplement: Table S3 — Description of the cohorts used for the analysis of SHANK copy-number variants. a,b Indicate the publications with overlap in the cohorts. * The total number of independent cases or controls is not the addition of the cohorts from each study due to the redundancy of the cases and controls tested from these publications. The independent numbers were obtained in collaboration with the authors of the corresponding publications (see Material & Method in supplementary Appendix). The parental DNA of controls was not available. PARIS, Paris Autism Research International Sibpair study; SSC, Simons Simplex Collection; AGP, Autism Genome Project; ACC, Autism Case Control; SAGE, Study of Addiction: Genetics and Environment; CHOP, Children's Hospital of Philadelphia; WTCCC2, Wellcome Trust Case Control Consortium; AGRE, Autism Genetic Resource exchange; NIMH, National Institute of Mental Health; SNP, Single Nucleotide Polymorphism; CEU, Utah residents with Northern and Western European ancestry from the CEPH collection; BAC, Bacterial Artificial Chromosome; CGH, Comparative Genomic Hybridization; ROMA, Representational Oligonucleotide Microarray Analysis; ADI-R, Autism Diagnostic Interview-Revised; ADOS, Autism Diagnostic Observation Schedule; DSM-IV-TR, Diagnostic and Statistical Manual of Mental Disorders, Fourth Edition-Text Revision; DISCO, Diagnostic Interview for Social and Communication Disorders; IQ, Intellectual Quotient; RPM, Raven's Progressive Matrices; PPVT, Peabody Picture Vocabulary Test. (DOC) [file pgen.1004580.s009.doc]

Table S3: Description of the Cohorts used for the analysis of *SHANK* Copy-number variants

| **Technologies** | **Studies** | **ASD** | | | | **Controls** | | |
| --- | --- | --- | --- | --- | --- | --- | --- | --- |
|  |  | N | **Cohorts** | **Main inclusion & exclusion criteria** |  | N | Cohorts | **Main inclusion & exclusion criteria** |
| SNP array (Illumina 1M-Duo) | This study^b^ | 306 | **PARIS study**: France, Sweden, Norway, Italy, Belgium, Austria, and the United States. **All samples:** 85% simplex; male-to-female ratio: 3.6:1; ethnicity: 70% Caucasian, 3.6% mixed, 1.3% African, 1.3% Asian, 23.8% unknown (based on self report). | **ASD:** ADIR, ADOS, DSM-IV-TR (In Sweden, for some cases, the DISCO-10 was applied instead of the ADI-R)  **IQ:** Wechsler, RPM, PPVT  **Exclusion:** syndromic autism (i.e. those with syndromes associated with severe mental retardation or other congenital anomalies), known cytogenetic abnormalities, Fragile X syndrome | | 454 | **France, Sweden**; **All samples:** male-to-female ratio: 1.05:1; ethnicity: 100% Caucasian | **France:** Healthy volunteers, interviewed with the DIGS and the FIGS to confirm the absence of both personal and family history of psychiatric disorders in first- and second-degree relatives.  **Sweden:** recruited in a study of obesity and body fat distribution; no known personal or familial history of ASD. |
| SNP array (Illumina IMv1 & IMv3) | Sanders *et al*. (2011) | 872 | **SSC (**simplex: 100%; male to female ratio was 6.2:1age at inclusion: 9.1 y (4–18 y); FSIQ: 85.1 (SD 1.5) (range <20–167); ethnicity: 79% Caucasian, 9% mixed, 4% African, 4% Asian, 4% others.) | **ASD:** ADIR, ADOS, DSM-IV-TR  **IQ:** Wechsler, RPM, PPVT | | 872 | **SSC** (872, unaffected sib; age at inclusion: 10.0 years (3.5–26 years)) | **SSC** No known personal history of ASD based on SRS |
| SNP array (Affymetrix 500K & 6.0, Illumina infinium 1M & 1M-Duo) & CGH array (Agilent 1M) | Pinto *et al*. (2010)^a^ | 996 | **AGP** (simplex: 53%) | **ASD: *Strict class***: criteria for autism based on ADIR+ ADOS; ***Broad class*:** criteria for autism based on ADIR and ADOS criteria for ASD, but not autism, or vice; ***Spectrum class*:** ASD on both the ADI-R and ADOS or who were not evaluated on one of the instruments but were diagnosed with autism on the other instrument  **IQ:** range of cognitive tests was administered, and standard scores were combined across tests to provide consolidated IQ estimates. | | 1 287 | **SAGE** (1,287; male to female ratio was 1:2, Age at inclusion: 39.2 (SD: 4.9) y, ethnicity: 100% Caucasian | **SAGE:** no known personal history of alcohol or drug dependence |
|  | Moessner *et al*. (2007)^a^ | 400 | The Hospital for Sick Children (225) and in child diagnostic centers in Hamilton, Ontario (100), and in St. John’s, Newfoundland (75). **All samples**: ethnicity: 100% Caucasian | **ASD:** ADIR, ADOS, DSM-IV-TR  **IQ:** Wechsler, RPM, PPVT  **Exclusion:** Fragile X syndrome | | 500 | **German PopGen** (500) | **German PopGen:** Living in Northern Schleswig-Holstein, Germany. Healthy control individuals are identified through official population registries and contacted by mail. |
|  | Marshall *et al*. (2008)^a^ | 427 | The Hospital for Sick Children (228), McMaster University (99), Memorial University (86), Others (14). **All samples:** simplex: 55%; ethnicity: 100% Caucasian | **ASD:** ADIR, ADOS, DSM-IV-TR  **IQ:** no information  **Exclusion:** Fragile X syndrome | | 1 652 | **German PopGen** (500)  **Ontario population-based controls Colon cancer study** (1,152)  **All samples:** ethnicity: 100% Caucasian | **German PopGen** (see above)  **Ontario Population Genomics Project:** living in Ontario, Canada. Recruited by telephone from a list of randomly selected residential telephone numbers for Ontario and from population-based Tax Assessment Rolls of the Ontario Ministry of Finance. |
|  | Berkel *et al*. (2010)^a^ | 396 | The Hospital for Sick Children, McMaster University. **All samples**: ethnicity: 100% Caucasian | **ASD:** ADIR, ADOS, DSM-IV-TR  **IQ:** Wechsler, RPM, PPVT  **Exclusion:** Fragile X syndrome | | 5 023 | **Ottawa Heart Genomics study** (1,234)  **German PopGen** (1,123)  **CEU Hapmap II** (59)  **SAGE** (1,287)  **CHOP pediatric control study** (1,320)  **All samples:** ethnicity: 100% Caucasian | **Ottawa Heart Genomics study**  **German PopGen** (see above)  **CEU Hapmap II**  **SAGE** (see above)  **CHOP pediatric control study** no known personal history of ASD; no chronic disease and are developmentally on target |
|  | Sato *et al*. (2012)^a,b^ | 1 614 | **Canadian cases**: (1,158) Hospital for Sick Children, Toronto, Ontario; McMaster University, Hamilton, Ontario; Memorial University of Newfoundland, St. John’s, Newfoundland; University of Alberta, Edmonton, Alberta; and the Montreal Children’s Hospital of the McGill University Health Centre, Montreal, Quebec.  **PARIS study: (**456) see above. **All samples**: ethnicity: 100% Caucasian | **ASD:** ADIR, ADOS, DSM-IV-TR (In Sweden, for some cases, the DISCO-10 was applied instead of the ADI-R)  **IQ:** Wechsler, RPM, PPVT  **Exclusion:** Fragile X syndrome | | 15 122 | **CHOP pediatric control study** (2,026)  **University of Washington** (2,493)  **German PopGen** (1,123)  **Ottawa Heart Genomics study** (1,234)  **Ontario population-based controls Colon cancer study** (1,120)  **HapMap III** (1,056)  **WTCCC2** (4,783)  **SAGE consortium** (1,287)  **All samples:** ethnicity: 100% Caucasian | **CHOP pediatric control study German PopGen, Ottawa Heart Genomics study, SAGE, Ontario Population Genomics Project** (see above)  **University of Washington:** Pharmacogenomics and Risk of Cardiovascular Disease (PARC), neurologically normal individuals identified at the National Institute for Neurological Disorders and Stroke (NINDS), and the Human Genome Diversity Panel (HGDP**)** |
| BAC-tilling CGH array (BAC 33K & 38K) & Oligonucleotide CGH array (Agilent 180K & 233K) | Bremer *et al*. (2010) | 223 | 25 cases were syndromic with an IQ within the normal range, 45 cases were syndromic and had MR, 60 cases were non syndromic with a normal IQ, and 93 patients were non syndromic but had ID. **All samples:** simplex: 87%; ethnicity: 100% Caucasian | **ASD:** ADIR, ADOS, DSM-IV-TR  **IQ:** Weschsler scales, Leiter | | 0 |  |  |
| SNP array (Illumina InfiniumII & HumanHap550) | Glessner *et al*. (2009) | 2 195 | **ACC** (859, male to female ratio was 4.5:1; Age at inclusion: 9.9 (SD: 7.9) y; FSIQ 87 (SD: 25.5); simplex: 55%; ethnicity: 100% Caucasian)  **AGRE** (1336, male to female ratio was 3.7:1; age at inclusion: 7.8 (SD: 4.4) y; FSIQ 100.7 (SD: 18.9); simplex: 5%) | **ACC:** all met the diagnostic criteria for autism on the basis of (ADI), and 124 met the criteria for other ASDs on the basis of (ADOS)  **AGRE**: patients were classified autism or broad spectrum (patterns of impairment along the spectrum, including PDD-NOS and Asperger syndrome)  **IQ:** Wechsler, RPM, PPVT | | 2 519 | **CHOP pediatric control study** (2519; male to female ratio was 1.1:1; age at inclusion: 8.7 y (SD :5.46); | **CHOP** See above |
| ROMA | Sebat *et al*. (2007) | 195 | **AGRE** (117), **NIMH** (38), **Skuse** (23), **Bregman** (9), **Giliam** (2). **All samples:** simplex: 61% | **ASD:** ADIR, ADOS, DSM-IV-TR  **IQ:** no information  **Exclusion Criteria:** syndromic autism (i.e. those with syndromes associated with severe mental retardation or other congenital anomalies) and to exclude known cytogenetic abnormalities. | | 196 | AGRE (31), NIMH (12), Skuse (20), Sutcliffe (13), Giliam (3), Chung (78), CEPH (19), Levy (1) | **AGRE, NIMH, Skuse, Sutcliffe**: no known family history of illness  **Other cohorts**: no known personal history of illness other than (a) obesity (Chung), (b) psychosis (Levy) or (c) Spinal Muscular Atrophy (Giliam) |
|  | **Total independent individuals*** | **5 657** |  |  | | **19 163** |  |  |
