# Supplementary material for: Meta-analysis of SHANK Mutations in Autism Spectrum Disorders: A Gradient of Severity in Cognitive Impairments
Source: PLoS Genet. 2014 Sep 4;10(9):e1004580. doi: 10.1371/journal.pgen.1004580 (PMC4154644; doi:10.1371/journal.pgen.1004580)
Supplement: Table S4 — Description of the cohorts used for the analysis of SHANK coding-sequence variants. a In this study, controls were tested using allelic discrimination by TaqMan technology and only for the variations identified in ASD. The parental DNA of controls was not available. SUVIMAX, Supplémentation en Vitamines et Minéraux Antioxydants; ADI-R, Autism Diagnostic Interview-Revised; ADOS, Autism Diagnostic Observation Schedule; DSM-IV-TR, Diagnostic and Statistical Manual of Mental Disorders, Fourth Edition-Text Revision; DISCO, Diagnostic Interview for Social and Communication Disorders; IQ, Intellectual Quotient; RPM, Raven's Progressive Matrices; PPVT, Peabody Picture Vocabulary Test; SSC, Simons Simplex Collection. (DOC) [file pgen.1004580.s010.doc]

Table S4: Description of the cohorts used for the analysis of *SHANK* coding-sequence variants

| **Genes** | **Studies** | **ASD** | | | **Controls** | | |
| --- | --- | --- | --- | --- | --- | --- | --- |
|  |  | N | **Cohorts** | **Main inclusion & exclusion criteria** | N | Cohorts | **Main inclusion & exclusion criteria** |
| ***SHANK1*** | This study | 251 | **PARIS study**: France, Sweden, Norway, Italy, Belgium, Austria, and the United States. All samples: 74.5% simplex; male-to-female ratio: 4.5:1; ethnicity: 92.8% Caucasian, 2.8% mixed, 2% African, 1.6% Asian, 0.8% unknown (based on self report). | **ASD:** ADIR, ADOS, DSM-IVTR (In Sweden, for some cases, the DISCO-10 was applied instead of the ADI-R)  **IQ:** Wechsler, RPM, PPVT  **Exclusion:** syndromic autism (i.e. those with syndromes associated with severe mental retardation or other congenital anomalies), known cytogenetic abnormalities, Fragile X syndrome | 492 | **France, Sweden;** (an additional cohort of 250 patients was sequenced for rare variants); Ethnicity: 100% Caucasian | **SUVIMAX Study:** controls recruited by a call for volunteers living in France (women aged 35-60 years or men aged 45-60 years) |
|  | Sato *et al*. (2012) | 509 | **Canadian case**: Hospital for Sick Children, Toronto, Ontario; McMaster University, Hamilton, Ontario; Memorial University of Newfoundland, St. John’s, Newfoundland; University of Alberta, Edmonton, Alberta; and the Montreal Children’s Hospital of the McGill University Health Centre, Montreal, Quebec. **PARIS study:** see above. All samples: ethnicity: 100% Caucasian | **ASD:** ADIR, ADOS, DSM-IVTR (In Sweden, for some cases, the DISCO-10 was applied instead of the ADI-R)  **IQ:** Wechsler, RPM, PPVT  **Exclusion:** Fragile X syndrome | 0^a^ | **Ontario Population Genomics Project** (285, TaqMan testing only for identified CSV); Ethnicity: 100% Caucasian | **Ontario Population Genomics Project:** living in Ontario, Canada. Recruited by telephone from a list of randomly selected residential telephone numbers for Ontario and from population-based Tax Assessment Rolls of the Ontario Ministry of Finance. |
| ***SHANK2*** | Leblond *et al*. (2012) | 455 | **PARIS study**: France, Sweden, Norway, Italy, Belgium, Austria, and the United States. All samples: 55.5% simplex; male-to-female ratio: 3.4:1; ethnicity: 56% Caucasian, 44% unknown (based on self report). | See above | 432 | **France, Sweden;** ethnicity: 100% Caucasian | **France:** Healthy volunteers, interviewed with the DIGS and the FIGS to confirm the absence of both personal and family history of psychiatric disorders in first- and second-degree relatives.  **Sweden:** recruited in a study of obesity and body fat distribution; no known personal or famlial history of ASD.  **Asthmatic cohort ?** |
|  | Berkel *et al*. (2010); | 396 | The Hospital for Sick Children, McMaster University. All samples: ethnicity: 100% Caucasian | **ASD:** ADIR, ADOS, DSM-IVTR  **IQ:** Wechsler, RPM, PPVT  **Exclusion:** Fragile X syndrome | 659 | **German PopGen** (374); **Ontario Population Genomics Project** (285); All subjects male to female ratio: 1.3:1; ethnicity: 100% Caucasian | **German PopGen** (see above)  **Ontario Population Genomics Project:** see above |
| ***SHANK3*** | This study | 429 | **PARIS study**: France, Sweden, Norway, Italy, Belgium, Austria, and the United States. All samples: 58.6% simplex; male-to-female ratio: 4.4:1; ethnicity: 54.7% Caucasian, 2.2% mixed, 2.4% African, 0.8% Asian, 39.8% unknown (based on self report). | See above | 0 |  | See above |
|  | Durand *et al*. (2007) | 227 | **PARIS study**: France, Sweden, Norway, Italy, Belgium, Austria, and the United States. All samples: 72% simplex; male to female ratio: 3.5:1; ethnicity: 91% Caucasian, 6% mixed, 2.5% African, 0.5% Asian | See above | 270 | **France** (120, age at inclusion: 19 to 65; male to female ratio: 1.1:1); **Sweden (**150, male to female ratio: 2.1:1); Ethnicity: 100% Caucasian | See above |
|  | Boccuto *et al*. (2012) | 325 | **South Carolina** **cohort** (237); **Italian cohort** (88); All samples: 92% simplex; male to female ratio: 3.9:1; ethnicity: 100% Caucasian | **ASD:** ADIR, ADOS, CARS  **IQ:** No information | 0 |  |  |
|  | Schaaf *et al*. (2011) | 339 | **SSC** (100% simplex; male to female ratio: 6.8:1; high functioning – FSIQ >70; ethnicity: 100% Caucasian) | **ASD:** ADIR, ADOS, DSM-IVTR  **IQ:** Wechsler, RPM, PPVT | 376 | **SSC** (male to female ratio: 6.8:1) | Exclusion if screening for depression, generalized anxiety disorder, alcohol dependence, drug dependence or obsessive compulsive behavior was positive |
|  | Gauthier *et al*. (2010) | 0 |  |  | 285 | **Canadian Cohort** (190, age at inclusion: 66 (SD 7.5) y; male to female ratio: 8.6:1); **French Cohort** (95); ethnicity: 99% Caucasian, 0.5% African, 0.5% Asian | 1/ recruited by advertisements in local newspapers  2/ Only individuals without any neuropsychiatric symptoms or family history of neuropsychiatric problems, including any psychotic symptoms, were included as negative controls (based on direct screening with the DIGS) |
|  | Gauthier *et al*. (2009) | 427 | **Canadian Cohort** (332); **French Cohort** (95); a All samples: age at inclusion: 12.3 y; male to female ratio: 6.4:1; ethnicity: 100% Caucasian | **ASD:** ADIR, ADOS, DSM-IVTR (the autism screening questionnaire (ASQ), a questionnaire derived from ADI-R, was administrated to all patients as an additional screening tool)  **IQ:** No information  **Exclusion:** syndromic autism (i.e. those with syndromes associated with severe mental retardation or other congenital anomalies), Fragile X syndrome | 190 | **Canadian Cohort** (190); ethnicity: 100% Caucasian | See above |
|  | Moessner *et al*. (2007) | 400 | The Hospital for Sick Children in Toronto (225); Child diagnostic centers in Hamilton, Ontario (100); St. John’s, Newfoundland (75). All samples: 38% simplex; ethnicity: 100% Caucasian | **ASD:** ADIR, ADOS, DSM-IVTR  **IQ:** Wechsler, RPM, PPVT  **Exclusion:** Fragile X syndrome | 100-200 | **HapMap sample;** ethnicity: 100% Caucasian |  |
