# Supplementary material for: Meta-analysis of SHANK Mutations in Autism Spectrum Disorders: A Gradient of Severity in Cognitive Impairments
Source: PLoS Genet. 2014 Sep 4;10(9):e1004580. doi: 10.1371/journal.pgen.1004580 (PMC4154644; doi:10.1371/journal.pgen.1004580)
Supplement: Table S5 — Prevalence of SHANK CNVs and coding-sequence variants in patients with ASD and controls. a Sato et al. (2012), Durand et al. (2007) Leblond et al. (2012) contained overlapping cohorts. For this study, the French and Swedish cases are in Leblond et al (2012) and the Canadian cases are in Sato et al. (2012). bThe controls from Sato et al. 2012 are not included here because they were only tested by Taqman for the variants identified in ASD. cThe two SHANK3 deletions reported by Glessner et al. (2009) in control subjects have not been validated and should be interpreted with caution. CSV, Coding-Sequence Variant; 95CI, 95% Confidence Interval. (DOC) [file pgen.1004580.s011.doc]

Table S5: Prevalence of *SHANK* CNVs and coding-sequence variants in patients with ASD and controls

|  |  |  | |  |  | **Number of patients - Frequency in ASD & 95CI (%)** | | | **Number of controls - Frequency in controls & 95CI (%)** | | |
| --- | --- | --- | --- | --- | --- | --- | --- | --- | --- | --- | --- |
|  |  | **Studies** | | **All patients with ASD** | **Controls** | ***SHANK1*** | ***SHANK2*** | ***SHANK3*** | ***SHANK1*** | ***SHANK2*** | ***SHANK3*** |
| **Copy-number variants** | | ALL *SHANKs* | This study + (Durand *et al.* 2007; Sato *et al.* 2012; Leblond *et al.* 2012)^a^ | 46 + 260 | 454 | 1 - 0.33[0.01-1.81] | 1 - 0.33[0.01-1.81] | 3 - 0.98[0.2-2.84] | 0 - 0[0-0.81] | 0 - 0[0-0.81] | 0 - 0[0-0.81] |
|  |  |  | Sanders *et al*. (2011) | 872 | 872 | 0 - 0[0-0,42] | 0 - 0[0-0,42] | 0 - 0[0-0,42] | 0 - 0[0-0.42] | 0 - 0[0-0.42] | 0 - 0[0-0.42] |
|  |  |  | Moessner *et al*. (2007); Marshall *et al*. (2008); Pinto *et al*. (2010); Berkel *et al*. (2010); Sato *et al*. (2012)^a^ | 1 866 | 15 122 | 1 - 0.05[0-0.3] | 2 - 0.11[0.01-0.39] | 3 - 0.16[0.03-0.47] | 0 - 0[0-0.02] | 0 - 0[0-0.02] | 0 - 0[0-0.02] |
|  |  |  | Bremer *et al*. (2010) | 223 | 0 | 0 - 0[0-1.64] | 0 - 0[0-1.64] | 1 - 0.45[0.01-2.47] | NA | NA | NA |
|  |  |  | Glessner *et al*. (2009) | 2,195 | 2 519 | 0 - 0[0-0.17] | 0 - 0[0-0.17] | 2 - 0.09[0.01-0.33] | 0 - 0[0-0.15] | 0 - 0[0-0.15] | 2 - 0.08[0.01-0.29]^c^ |
|  |  |  | Sebat *et al*. (2007) | 195 | 196 | 0 - 0[0-1.87] | 0 - 0[0-1.87] | 1 - 0.51[0.01-2.82] | 0 - 0[0-1.86] | 0 - 0[0-1.86] | 0 - 0[0-1.86] |
|  |  |  | **Total** | **5 657** | **19 163** | **2 - 0.04[0-0.13]** | **3 - 0.05[0.01-0.15]** | **10 - 0.18[0.08-0.32]** | **0 - 0[0-0.02]** | **0 - 0[0-0.02]** | **2 - 0.01[0-0.04]** |
| **Truncating CSV** | | *SHANK1* | This study | 251 | 492 | 0 - 0[0-1.46] |  |  | 0 - 0[0-0.75] |  |  |
|  |  |  | Sato *et al*. (2012) | 509 | 0^b^ | 0 - 0[0-0.72] |  |  | NA |  |  |
|  |  |  | **Total** | **760** | **492** | **0 - 0[0-0.48]** |  |  | **0 - 0[0-0.75]** |  |  |
|  |  | *SHANK2* | Leblond *et al*. (2012) | 455 | 432 |  | 0 - 0[0-0.81] |  |  | 0 - 0[0-0.85] |  |
|  |  |  | Berkel *et al*. (2010) | 396 | 659 |  | 1 - 0.25[0.01-1.40] |  |  | 0 - 0[0.56] |  |
|  |  |  | **Total** | **851** | **1 091** |  | **1 - 0.12[0-0.65]** |  |  | **0 - 0[0.34]** |  |
|  |  | *SHANK3* | This study + Durand *et al.* (2007) | 429 + 227 | 270 |  |  | 9 - 1.37[0.63-2.59] |  |  | 0 - 0[0-1.36] |
|  |  |  | Boccuto *et al*. (2012) | 325 | 0 |  |  | 1 - 0.31[0.01-1.70] |  |  | NA |
|  |  |  | Schaff *et al*. (2011) | 339 | 376 |  |  | 0 - 0[0-1.08] |  |  | 0 - 0[0-0.98] |
|  |  |  | Gauthier *et al*. (2009, 2010)) | 427 | 285 |  |  | 1 - 0.23[0.01-1.30] |  |  | 0 - 0[0-1.29] |
|  |  |  | Moessner *et al*. (2007) | 400 | 100-200 |  |  | 0 - 0[0-0.92] |  |  | 0 - 0[3.62] |
|  |  |  | **Total** | **2 147** | **1 031** |  |  | **11 - 0.51[0.26-0.91]** |  |  | **0 - 0[0-0.36]** |
