# Supplementary material for: Meta-analysis of SHANK Mutations in Autism Spectrum Disorders: A Gradient of Severity in Cognitive Impairments
Source: PLoS Genet. 2014 Sep 4;10(9):e1004580. doi: 10.1371/journal.pgen.1004580 (PMC4154644; doi:10.1371/journal.pgen.1004580)
Supplement: Table S6 — Prevalence of SHANK CNVs and coding-sequence variants in ASD patients with or without ID. a Sato et al. (2012) and Leblond et al. (2012) contained overlapping cohorts. For this study, the French and Swedish cases are in Leblond et al (2012) and the Canadian cases are in Sato et al. (2012). b The controls from Sato et al. 2012 are not included here because they were only tested by Taqman for the variants identified in ASD. CSV, Coding-Sequence Variant. (DOC) [file pgen.1004580.s012.doc]

Table S6: Prevalence of *SHANK* CNVs and coding-sequence variants in ASD patients with or without ID

|  |  |  |  |  | | | **Number of patients - Frequency & 95CI (%)** | | | **Number of patients - Frequency & 95CI (%)** | | |
| --- | --- | --- | --- | --- | --- | --- | --- | --- | --- | --- | --- | --- |
|  |  |  |  |  | | | **ASD with IQ<70** | | | **ASD with IQ>70** | | |
|  |  | **Studies** | **All patients with ASD** | **ASD Patients with IQ data available** | **IQ<70** | **IQ>70** | ***SHANK1*** | ***SHANK2*** | ***SHANK3*** | ***SHANK1*** | ***SHANK2*** | ***SHANK3*** |
| **Copy-number variants** | ALL *SHANKs* | This study + (Durand *et al.* 2007; Sato *et al.* 2012; Leblond *et al.* 2012)^a^ | 306 | 251 | 166 | 85 | 0 - 0[0-2.2] | 1 - 0.60[0.02-3.31] | 3 - 1.81[0.37-5.19] | 1 - 1.18[0.03-6.38] | 0 - 0[0-4.25] | 0 - 0[0-4.25] |
|  |  | Sanders *et al*. (2011) | 872 | 872 | 232 | 640 | 0 - 0[0-1.58] | 0 - 0[0-1.58] | 0 - 0[0-1.58] | 0 - 0[0-0.57] | 0 - 0[0-0.57] | 0 - 0[0-0.57] |
|  |  | Moessner *et al*. (2007); Marshall *et al*. (2008); Pinto *et al*. (2010); Berkel *et al*. (2010); Sato *et al*. (2012)^a^ | 1 866 | 1 432 | 519 | 913 | 0 - 0[0-7.1] | 2 - 0.39[0.05-1.39] | 3 - 0.58[0.12-1.68] | 1 - 0.11[0-0.61] | 0 - 0[0-0.40] | 0 - 0[0-0.40] |
|  |  | Bremer *et al*. (2010) | 223 | NA |  |  |  |  |  |  |  |  |
|  |  | Glessner *et al*. (2009) | 2 195 | NA |  |  |  |  |  |  |  |  |
|  |  | Sebat *et al*. (2007) | 195 | NA |  |  |  |  |  |  |  |  |
|  |  | **Total** | **5 657** | **2 555** | **917** | **1 638** | **0 - 0[0-0.40]** | **3 - 0.33[0.077-0.95]** | **6 - 0.65[0.24-1.42]** | **2 - 0.12[0.01-0.44]** | **0 - 0[0-0.22]** | **0 - 0[0-0.22]** |
| **Truncating CSV** | *SHANK1* | This study | 251 | 251 | 179 | 72 | 0 - 0[0-2.04] |  |  | 0 - 0[0-4.99] |  |  |
|  |  | Sato *et al*. (2012) | 509 | 381 | 99 | 282 | 0 - 0[0-3.66] |  |  | 0 - 0[0-4.40] |  |  |
|  |  | **Total** | **760** | **632** | **278** | **354** | **0 - 0[0-1.32]** |  |  | **0 - 0[0-1.04]** |  |  |
|  | *SHANK2* | Leblond *et al*. (2012) | 455 | 328 | 221 | 107 |  | 0 - 0[0-1.66] |  |  | 0 - 0[0-3.39] |  |
|  |  | Berkel *et al*. (2010) | 396 | 351 | 123 | 228 |  | 0 - 0[0-2.95] |  |  | 1 - 0.44[0.01-2.42] |  |
|  |  | **Total** | **851** | **679** | **344** | **335** |  | **0 - 0[0-1.07]** |  |  | **1 - 0.30[0-1.10]** |  |
|  | *SHANK3* | This study + Durand *et al.* (2007) | 656 | 656 | 538 | 118 |  |  | 9 - 1.67[0.77-3.15] |  |  | 0 - 0[0-3.08] |
|  |  | Boccuto *et al*. (2012) | 325 | NA |  |  |  |  |  |  |  |  |
|  |  | Schaff *et al*. (2011) | 339 | 339 | 0 | 339 |  |  | NA |  |  | 0 - 0[0-1.08] |
|  |  | Gauthier *et al*. (2009, 2010)) | 427 | NA |  |  |  |  |  |  |  |  |
|  |  | Moessner *et al*. (2007) | 400 | 283 | 73 | 210 |  |  | 0 - 0[0-4.93] |  |  | 0 - 0[1.74] |
|  |  | **Total** | **2 147** | **1 278** | **611** | **667** |  |  | **9 - 1.47[0.68-2.78]** |  |  | **0 - 0[0-0.55]** |
|  |  | **Total CNV + CVS** |  |  |  |  | **0** | **0.33** | **2.12** | **0.12** | **0.30** | **0** |
