# Supplementary material for: Meta-analysis of SHANK Mutations in Autism Spectrum Disorders: A Gradient of Severity in Cognitive Impairments
Source: PLoS Genet. 2014 Sep 4;10(9):e1004580. doi: 10.1371/journal.pgen.1004580 (PMC4154644; doi:10.1371/journal.pgen.1004580)
Supplement: Table S7 — Genetic and clinical features of ASD patients carrying de novo/deleterious SHANK mutations. a Mother showed anxiety and shyness. b Mutations not present in the mother and father not tested (DNA unavailable). c Father had a balanced translocation t(14;22)(p11.2;q13.33). d Mother had a balanced translocation t(14;22)(p11.2;q13.33). e No epilepsy, but abnormal EEG with bilateral epileptiform discharges. f Relatives of proband IV-1. g Case reports not reported in a systematic screening of SHANK2 or whole genome analysis. h Patient detected in an additional mutation screening of SHANK3 exon 21. i Relatives of AU016_3. j Not included in the figure: large deletion involving numerous genes or complex chromosomal rearrangement. k For SHANK3, we only included in figure 2 the patients from the PARIS cohort. l No pictures available. m The phenotypic features were characteristic to the Phelan-Mcdermid syndrome. AS, absence seizures; ASD, autism spectrum disorder; Asp, Asperger syndrome; Aut, autism; CNV, copy number variant, CSV, coding-sequence variants; del, deletion; f, female; ID; inh, inherited; m, male; U, unknown; y, years; GTCS, generalized tonic-clonic seizures. (DOC) [file pgen.1004580.s013.doc]

Table S7: Genetic and clinical features of ASD patients carrying *de novo*/deleterious *SHANK* mutations

| **Patient ID** | **CNV (size) or CSV** | **Genetic Abnormalities** | **Inheritance** | **Sex** | **ASD** | **Intellectual disability** | **Epilepsy (type, onset)** | **Included for the calculation of the prevalence** | **Reported in Figure 4** | **Reported in Table 2** | **References** |
| --- | --- | --- | --- | --- | --- | --- | --- | --- | --- | --- | --- |
| ***SHANK1*** |  |  |  |  |  |  |  |  |  |  |  |
| family 1, III-5 | CNV_del (63.8 kb) | Loss of exon 1 to 20 | inh (mother) **^a^** | m | Asp | 0 | 0 | 0 **^f^** | 1 | 0 **^l^** | Sato et al. 2012 |
| family 1, IV-1 | CNV_del (63.8 kb) | Loss of exon 1 to 20 | inh (mother) **^a^** | m | Asp | 0 | 0 | 1 | 1 | 0 **^l^** | Sato et al. 2012 |
| family 1, IV-3 | CNV_del (63.8 kb) | Loss of exon 1 to 20 | inh (mother) **^a^** | m | Asp | 0 | 0 | 0 **^f^** | 1 | 0 **^l^** | Sato et al. 2012 |
| family 2, II-1 | CNV_del (63.4 kb) | Loss of the last 3 exons of SHANK1 and all *SYT3 exons* | de novo | m | ASD | 0 | 0 | 1 | 1 | 0 **^l^** | Sato et al. 2012 |
| ***SHANK2*** |  |  |  |  |  |  |  |  |  |  |  |
| SK0441-003 | CSV | R841X (also named R462X) | *de novo* | m | ASD | no | 0 | 1 | 1 | 0 **^l^** | Berkel et al. 2010 |
| SK0217-003 | CNV_del (66 kb) | Loss of exon 6 and 7 | *de novo* | m | Aut | 1 (mild) | 0 | 1 | 1 | 1 | Pinto et al. 2010; Berkel et al. 2010 |
| 6319_3 | CNV_del (68 kb) | Loss of exon 14 and 15 | *de novo* | m | Aut | 1 (mild) | 0 | 1 | 1 | 1 | Pinto et al. 2010 |
| AU038_3 | CNV_del (421 kb) | Loss of exon 5 to 16 | *de novo* | m | Aut | 1 (moderate) | 0 | 1 | 1 | 1 | Leblond et al. 2012 |
| Wischmeijer et al. 2010 | CNV_del (3.4 Mb) | del_11q13.2q13.4 (all *SHANK2* exons) | *de novo* | f | Aut | 1 (severe) | 0 | 0 **^g^** | 0 **^j^** | 1 | Wischmeijer et al. 2010 |
| RDB_30769 | CNV_del (1.8 Mb) | del_11q13.3q13.4 (all *SHANK2* exons) | *de novo* | m | Aut | 1 (severe) | 0 | 0 **^g^** | 0 **^j^** | 1 | This study |
| AUL_001 | Translocation | t(1;7;11)(p35;q33;q12)dn; breakpoint in *SHANK2 (intron 14)* | *de novo* | m | Aut | 1 (severe) | 0 | 0 **^g^** | 0 **^j^** | 1 | This study |
| ***SHANK3*** |  |  |  |  |  |  |  |  |  |  |  |
| AUN_001 | CSV | P1005Rfs*73 | U **^b^** | f | Aut | 1 (severe) | 0 | 1 | 1 | 1 | This study |
| AUN_002 | CSV | G1339Efs*5 | U **^b^** | m | Aut | 1 (severe) | 1 (GTCS/8y) | 1 | 1 | 1 | This study |
| AUN_003 | CSV | E809X | *de novo* | f | Aut | 1 (moderate) | 0 | 1 | 1 | 1 | This study |
| AUN_004 | CSV | S1202Cfs*81 | *de novo* | m | Aut | 1 (moderate) | 0 | 1 | 1 | 1 | This study |
| AUN_005 | CSV | R1255Lfs*25 | *de novo* | m | Aut | 1 (moderate) | 1 (AS/U) | 1 | 1 | 1 | This study |
| AU285_4 | CSV | Q1243X | *de novo* | m | Aut | 1 (severe) | 0 | 0 **^h^** | 1 | 1 | This study |
| AU084_4 | CSV | A1076Efs*218 | *de novo* | m | Aut | 1 (moderate) | 0 | 1 | 1 | 0 **^l^** | This study |
| AU921_3 | CSV | L1142Vfs*153 | *de novo* | m | Aut | 1 (severe) | 1 (GTCS/10y) | 1 | 1 | 1 | This study |
| AU380_3 | CSV | G1271Af*15 | *de novo* | m | Aut | 1 (severe) | 0 | 1 | 1 | 0 **^l^** | This study |
| AU016_3; ASD2 | CSV | A1227Gfs*69 | *de novo* | m | Aut | 1 (moderate) | 0 | 1 | 1 | 0 **^l^** | Durand et al. 2007 |
| AU016_5; ASD2 | CSV | A1227Gfs*69 | *de novo* | m | Aut | 1 (moderate) | 1 (GTCS/17y) | 0 **^i^** | 1 | 0 **^l^** | Durand et al. 2007 |
| SK007-ASD4 | CSV | Q321R | *de novo* | f | ASD | 1 (U) | 0 ^e^ | 1 | 0 **^k^** | 0 **^l^** | Moessner et al. 2007 |
| Gauthier et al 2008 | CSV | S755Sfs*1 | *de novo* | m | ASD | 1 (U) | U | 1 | 0 **^k^** | 0 **^l^** | Gauthier et al 2008 |
| CMS14055 | CSV | E1295Rfs*90 | U **^b^** | m | ASD | 1 (severe) | 1 (U/0.8y) | 1 | 0 **^k^** | 0 **^l^** | Boccuto et al. 2012 |
| 12718 | CSV | P141A | *de novo* | f | Aut | 1 (U) | 1 (U/U) | 1 | 0 **^k^** | 0 **^l^** | Boccuto et al. 2012 |
| AU029_4 | CNV_del (1.5 Mb) | del_22q13.32q13.33 (all *SHANK3* exons) | *de novo* | f | Aut | 1 (severe) | 0 | 1 | 1 | 0 **^m^** | This study |
| AUN_006 | CNV del (?) | Breakpoint in intron 8 of *SHANK3* | *de novo* | m | Aut | 1 (severe) | U | 0 | 0 | 0 | This study |
| AUN_007 | CNV del (?) | Breakpoint in exon 22 | *de novo* | m | Aut | 1 (severe) | 0 | 0 | 1 | 0 | This study |
| AU112_3; ASD1 | CNV_del (142 kb) | Breakpoint in intron 8 of *SHANK3* | *de novo* | m | Aut | 1 (severe) | 0 | 1 | 1 | 0 **^m^** | Durand et al. 2007 |
| ASD3 | CNV_del (800 kb) | 22qter monosomy (all *SHANK3* exons) | *de novo* **^c^** | f | Aut | 1 (U) | 0 | 1 | 1 | 0 **^m^** | Durand et al. 2007 |
| 89-3524-100 | CNV_del (4.3 Mb) | del_22q13.31-q13.33 (all *SHANK3* exons) | *de novo* **^d^** | f | Aut | 1 (U) | U | 1 | 0 **^k^** | 0 **^m^** | Sebat et al. 2007 |
| case 11 | CNV_del (25kb) | del_22q13.33 (all *SHANK3* exons) | *de novo* | f | Aut | 1 (U) | U | 1 | 0 **^k^** | 0 **^m^** | Bremer et al. 2010 |
| ACC case 1 | CNV_del (U) | del_22q13.33 | U | U | Aut | 1 (U) | U | 1 | 0 **^k^** | 0 **^m^** | Glessner et al. 2009 |
| ACC case 2 | CNV_del (U) | del_22q13.33 | U | U | Aut | 1 (U) | U | 1 | 0 **^k^** | 0 **^m^** | Glessner et al. 2009 |
| 3524 | CNV_del (4.4 Mb) | del_22q13.31-q13.33 (all *SHANK3* exons) | *de novo* | m | Aut | 1 (U) | 1 (U/U) | 1 | 0 **^k^** | 0 **^m^** | Moessner et al. 2007 |
| NA0039-000 | CNV_del (3.2 Mb) | del_22q13.31-q13.33 (all *SHANK3* exons) | *de novo* **^c^** | f | Aut | 1 (severe) | 0 | 1 | 0 **^k^** | 0 **^m^** | Moessner et al. 2007; Marshall et al. 2008 |
| MM0109-003 | CNV_del (277 kb) | del_22q13.33 (all *SHANK3* exons) | *de novo* | f | Aut | 1 (severe) | U | 1 | 0 **^k^** | 0 **^m^** | Moessner et al. 2007; Marshall et al. 2008 |
