# Supplementary material for: Meta-analysis of SHANK Mutations in Autism Spectrum Disorders: A Gradient of Severity in Cognitive Impairments
Source: PLoS Genet. 2014 Sep 4;10(9):e1004580. doi: 10.1371/journal.pgen.1004580 (PMC4154644; doi:10.1371/journal.pgen.1004580)
Supplement: Table S8 — Primers used for mutation screening of SHANK1 and SHANK3. The red sequences correspond to the M13 adaptor (M13F = TGTAAAACGACGGCCAGT & M13R = GGATAACAATTTCACACAGG). PCR, Polymerase Chain Reaction; TpQ, Tampon Q (from Qiagen); DMSO, Dimethyl sulfoxide. (DOC) [file pgen.1004580.s014.doc]

Table S8. Primers used for mutation screening of *SHANK1* and *SHANK3*

| **Amplicon name** | **Exon** | **Size (bp)** | **PCR Forward primer (5'-3')** | **PCR Reverse primer (5'-3')** | **Annealing** | **Sequence Primer** |
| --- | --- | --- | --- | --- | --- | --- |
| SHANK1_E1E2 | E1 & E2 | 940 | GCCTCCTTCCTGCCTATCTT | AAGGTCTGGACTCGCACACT | 62°C | Both PCR primers |
| SHANK1_E3 | E3 | 394 | CCATCTACCGCCTAGACCAA | GGTACAGCATCCCAAGTTAAG | 57°C | Forward PCR primer |
| SHANK1_E4E5 | E4 & E5 | 750 | CTGACTGTCCTGTCTTCTCTC | GGTCTGTGTGATGTCAGCTAT | 60°C | Reverse PCR primer & CCAGTCAGGAATGGATGTGA |
| SHANK1_E6E7 | E6 & E7 | 1300 | TTTCAATGGCTCATCCAAGA | GGCCATGAGCTCAACACTTT | 58°C | Forward PCR primer & GATGCGGTGAACGATAGGAT |
| SHANK1_E8E9 | E8 & E9 | 708 | CTAAAGTGCTCCTGGTCCGTA | CCACCACCATCCAGACATTT | 62°C | Both PCR primers |
| SHANK1_E10 | E10 | 518 | CTCTCCCACCATCTCTTGCCC | ACAGAGGAAGGCAGCTGGGC | 60°C; TpQ 10% | Forward PCR primer |
| SHANK1_E11 | E11 | 577 | CTCGTGAGCGGAGGGATGAC | ATGGTCTGCTCTCTGCTCCAC | 60°C; TpQ 10% | Reverse PCR primer |
| SHANK1_E12E13 | E12 & E13 | 567 | GAATGCTCTAAGTTGAGTGTG | ACTTGAGAAGGAACAGATTAG | 54°C | Both PCR primers |
| SHANK1_E14 | E14 | 303 | GGAGGCCCTTGTCTCCTTCAT | GAAACCTCAGCTCTGGTCGTG | 60°C; TpQ 10% | Forward PCR primer |
| SHANK1_E15E16 | E15 & E16 | 667 | CACAGAGTGGGTGACAGAGAT | CAGATCCTGGTGTGAATCATG | 54°C; TpQ 10% | Both PCR primers |
| SHANK1_E17E18 | E17 & E18 | 622 | GGTCTCTGAAGCTGCAAGCAC | AACGAACTCAAGGGATGGTC | 60°C | TCCCAGGGCTCAGAGAATAG & CCTAGCACTGCTTCTCCGTG |
| SHANK1_E19E20 | E19 & E20 | 665 | CTGGGCGTCATCCGTGTTCTT | CAGGTTGGTGTGTGAACCGC | 60°C | GAGATTGTGTCTCCAAGCGG & CCGCTTGGAGACACAATCTC |
| SHANK1_E21 | E21 | 268 | AGTGAAGCCACAAGCCTTTC | CACAGAACATTTCCAAGTCTG | 52°C | Reverse PCR primer |
| SHANK1_E22_A | E22 | 790 | TGCAGTGCACAACCTGTACC | GGCAGCTGGAAATAGCGTAG | Touchdown; TpQ 20%; DMSO 5% | Both PCR primers |
| SHANK1_E22_B | E22 | 841 | CTCCCGAGATGGAGACAGG | GACTCCAGTCGGAGGTAGGG | Touchdown; DMSO 5% | Both PCR primers |
| SHANK1_E22_C | E22 | 973 | CTGTTCCTGTCCACCGACG | GCTTTTCGAAGCTGTTGGAG | Touchdown; DMSO 5% | Both PCR primers |
| SHANK1_E22_D | E22 | 672 | AGGGCCAGCGAAGAGAAC | CCGGAGCTTAGAGGGAGTC | Touchdown; DMSO 5% | Both PCR primers |
| SHANK1_E22_E | E22 | 779 | AGCCTATCTGCCGAAGGTG | CCAACCTGGTTTCTGTTTCC | Touchdown; DMSO 5% | Both PCR primers |
| SHANK1_E23 | E23 | 1218 | TTACCCCTTGCTCCCTCCATT | CTCAGGGCTGACCCTCTAT | 61°C | CTGCCTTCCGGACCCCTCTA & CAATCAGCCACGTCGAACTT |
| SHANK3_E1 | E1 | 323 | TGTAAAACGACGGCCAGTGCGCTCCGTTCCCCGGCGCGA | GGATAACAATTTCACACAGGCCTCCGCGAACCGCGGCCGAA | Touchdown | M13F & M13R |
| SHANK3_E2 | E2 | 320 | TGTAAAACGACGGCCAGTGACCTGAGCTCACGAGCCCGCT | GGATAACAATTTCACACAGGCTGCCGTGCCCTTCACTGGTC | Touchdown | M13F & M13R |
| SHANK3_E3 | E3 | 326 | TGTAAAACGACGGCCAGTTGCGGTGTGGCCAGCATGAG | GGATAACAATTTCACACAGGTCAGCCACACCCAGTACAGGCTCTG | Touchdown | M13F & M13R |
| SHANK3_E4E5 | E4 & E5 | 595 | TGTAAAACGACGGCCAGTTGGGAGTGCAGGACCGTGGTTGAC | GGATAACAATTTCACACAGGTGAGCAGCCTCAGTATCCACACCAG | Touchdown | M13F & M13R |
| SHANK3_E6E7 | E6 & E7 | 593 | TGTAAAACGACGGCCAGTTCTTGCCTGGTGATGGGGCTGG | GGATAACAATTTCACACAGGGCACACATGCAATCACCGTACAGG | Touchdown | M13F & M13R |
| SHANK3_E8 | E8 | 318 | TGTAAAACGACGGCCAGTTGTGAGTCCGTGTGTGTGAGCCTG | GGATAACAATTTCACACAGGGTGCTCCCTTAGGGCCTTCCAGGGA | Touchdown | M13F & M13R |
| SHANK3_E9 | E9 | 252 | TGTAAAACGACGGCCAGTACGAACTGGAGAAGGAGCAG | GGATAACAATTTCACACAGGCACCACTGACCCCCACATCT | Touchdown | M13F & M13R |
| SHANK3_E10 | E10 | 509 | TGTAAAACGACGGCCAGTGTTTGCCTGGAACCAAGGCAGTTCC | GGATAACAATTTCACACAGGTAGGCACCACCAGGCCTCTC | Touchdown | M13F & M13R |
| SHANK3_E11 | E11 | 332 | TGTAAAACGACGGCCAGTGGCATCGCGTCCGTCACCTACGT | GGATAACAATTTCACACAGGGAGGAGACCCCAGAGCCACTG | Touchdown | M13F & M13R |
| SHANK3_E12 | E12 | 248 | TGTAAAACGACGGCCAGTTGGGCAGAACCTGCTCCTGAGGTG | GGATAACAATTTCACACAGGAACTGGAAGGGGTGGTCCCCTTGG | Touchdown | M13F & M13R |
| SHANK3_E13E14 | E13 & E14 | 548 | TGTAAAACGACGGCCAGTTGTGTGGGCAGAGACTGGTGACC | GGATAACAATTTCACACAGGGACATCCCTGAGCGGTGTGCA | Touchdown | M13F & M13R |
| SHANK3_E15E16 | E15 & E16 | 584 | TGTAAAACGACGGCCAGTCCACCCGAACCTAGCTGGTGAAGC | GGATAACAATTTCACACAGGGTCTAGCACCAGGGATCGGGA | Touchdown | M13F & M13R |
| SHANK3_E17 | E17 | 287 | TGTAAAACGACGGCCAGTTGGAGCGCAAGTGGCACCTGCA | GGATAACAATTTCACACAGGCTGTCCTTCCTACCCTCTGGCTGGA | Touchdown | M13F & M13R |
| SHANK3_E18 | E18 | 285 | TGTAAAACGACGGCCAGTGGCAGATTTGCTATTCACGG | GGATAACAATTTCACACAGGGCTGGAACCTCCTCACACAC | Touchdown | M13F & M13R |
| SHANK3_E19 | E19 | 330 | TGTAAAACGACGGCCAGTGGTTGGGGAGGACATGGCAGTG | GGATAACAATTTCACACAGGCATGGGGTGCACACACCCCTCTGGA | Touchdown | M13F & M13R |
| SHANK3_E20 | E20 | 307 | TGTAAAACGACGGCCAGTCTAGTGCCATTGGAGTGAGAGCGTG | GGATAACAATTTCACACAGGCTGGAAACCCCAGTTATGGGCAGAG | Touchdown | M13F & M13R |
| SHANK3_E21_A | E21 | 634 | TGTAAAACGACGGCCAGTTTGTGTCCGGACGGTGGCTTCC | GGATAACAATTTCACACAGGACGGAGCGAAGAGGCTGGCGCTGAA | Touchdown | M13F & M13R |
| SHANK3_E21_B | E21 | 634 | TGTAAAACGACGGCCAGTGCGCTCCATGATCATCCTGCA | GGATAACAATTTCACACAGGTTTGCCGGTGAGTGGGTGGA | Touchdown | M13F & M13R |
| SHANK3_E21_C | E21 | 618 | TGTAAAACGACGGCCAGTGCGGATCTGCCATCCCTACAGC | GGATAACAATTTCACACAGGCACAGCCGCTGACTGCATGG | Touchdown | M13F & M13R |
| SHANK3_E21_D | E21 | 626 | TGTAAAACGACGGCCAGTGCTGGCCTCATCGTTGTGCACGCCA | GGATAACAATTTCACACAGGTTGGGAGGCACTGGTGGCTTCTCGA | Touchdown | M13F & M13R |
| SHANK3_E21_E | E21 | 698 | TGTAAAACGACGGCCAGTAGGCTGACACACGCAGCTCC | GGATAACAATTTCACACAGGAGGCCAAGCAAGACCGGATTCAG | Touchdown | M13F & M13R |
| SHANK3_E22_A | E22 | 604 | TGTAAAACGACGGCCAGTTTCCTCTGGCCGGGCTACTC | GGATAACAATTTCACACAGGCATGGTCCTCGAAGCGGTCG | Touchdown | M13F & M13R |
| SHANK3_E22_B | E22 | 468 | TGTAAAACGACGGCCAGTTTCGTGGTGCGCAGCGTGAG | GGATAACAATTTCACACAGGCTCCGAGCAACAGCAAACAGGACGA | Touchdown | M13F & M13R |
