# Supplementary material for: Meta-analysis of SHANK Mutations in Autism Spectrum Disorders: A Gradient of Severity in Cognitive Impairments
Source: PLoS Genet. 2014 Sep 4;10(9):e1004580. doi: 10.1371/journal.pgen.1004580 (PMC4154644; doi:10.1371/journal.pgen.1004580)
Supplement: Table S9 — SHANK1 coding-sequence variants identified in 760 patients with ASD and 492 controls. aNucleotide positions are according to NM_016148 from NCBI37/hg19 on the positive DNA strand (chromosome 19). The patients with ASD used for this analysis came from this study (n = 240) and from the study of Sato et al. (2012) (n = 509). The Grantham matrix and GERP scores were obtained from SeattleSeq Annotation 134. We used the Fisher's exact test (2-sided) and Pearson's Chi-squared test with Yates' continuity correction. P, p-value; ASD, Autism Spectrum Disorder; MAF, Minor Allele Frequency; GERP, Genomic Evolutionary Rate Profiling; pph2_class, polyphen-2_class. (DOC) [file pgen.1004580.s015.doc]

Table S9: *SHANK1* coding-sequence variants identified in 760 patients with ASD and 492 controls

|  | Detected variants | | | Frequency | | GERP | Grantham | pph2_class | Protein domain | Study |
| --- | --- | --- | --- | --- | --- | --- | --- | --- | --- | --- |
|  | Exon | Nucleotide/dbSNP^a^ | Amino Acid | ASD (n=760) | Controls (n=492) |  |  | HumDiv |  |  |
| ASD only | 1 | g.51220161C>T | p.A6T | 1 | 0 | 3.180 | 58 | neutral | SPN domain | This study |
|  | 1 | g.51220076C>T | p.G34D | 1 | 0 | 2.650 | 94 | deleterious | SPN domain | Sato *et al*. (2012) |
|  | 1 | g.51219998C>T | p.R60H | 1 | 0 | 3.210 | 29 | neutral | SPN domain | Sato *et al*. (2012) |
|  | 6 | g.51215287C>T | p.D293N | 2 | 0 | 4.680 | 23 | deleterious | - | Sato *et al*. (2012) |
|  | 10 | g.51206988G>T | p.T441N | 1 | 0 | 3.130 | 65 | neutral | - | Sato *et al*. (2012) |
|  | 10 | g.51206952G>A | p.A453V | 1 | 0 | 4.280 | 64 | deleterious | - | This study |
|  | 11 | g.51205886C>T | p.G529R | 1 | 0 | 4.080 | 125 | deleterious | - | Sato *et al*. (2012) |
|  | 11 | g.51205840C>T | p.R544H | 1 | 0 | 3.170 | 29 | deleterious | - | This study |
|  | 17 | g.51191281C>T | p.R736Q | 1 | 0 | 3.330 | 43 | deleterious | PDZ domain | Sato *et al*. (2012) |
|  | 22 | g.51172180G>A | p.P1013S | 1 | 0 | 0.443 | 74 | neutral | Proline rich region | Sato *et al*. (2012) |
|  | 22 | g.51171270C>T | p.G1316D | 2 | 0 | 1.500 | 94 | deleterious | Proline rich region | Sato *et al*. (2012) |
|  | 22 | g.51170856C>T | p.G1454E | 1 | 0 | 1.640 | 98 | neutral | Proline rich region | Sato *et al*. (2012) |
|  | 22 | g.51170854C>T | p.V1455M | 1 | 0 | 1.640 | 21 | deleterious | Proline rich region | Sato *et al*. (2012) |
|  | 22 | g.51170826G>A | p.T1464M | 1 | 0 | 1.640 | 81 | neutral | Proline rich region | This study |
|  | 22 | g.51170779C>T | p.A1480T | 1 | 0 | 1.780 | 58 | neutral | Proline rich region | Sato *et al*. (2012) |
|  | 22 | g.51170775G>A | p.A1481V | 2 | 0 | 0.427 | 64 | neutral | Proline rich region | Sato *et al*. (2012), this study |
|  | 22 | g.51170674C>A | p.G1515W | 1 | 0 | 2.000 | 184 | deleterious | Proline rich region | Sato *et al*. (2012) |
|  | 22 | g.51170418G>A | p.T1600I | 1 | 0 | 1.290 | 89 | neutral | Proline rich region | Sato *et al*. (2012) |
|  | 22 | g.51170407G>T | p.P1604T | 1 | 0 | -0.294 | 38 | neutral | Proline rich region | Sato *et al*. (2012) |
|  | 22 | g.51170362A>T | p.S1619T | 1 | 0 | 1.140 | 58 | deleterious | Proline rich region | Sato *et al*. (2012) |
|  | 22 | g.51170359T>C | p.T1620A | 1 | 0 | 1.310 | 58 | deleterious | Proline rich region | Sato *et al*. (2012) |
|  | 22 | g.51170046A>T | p.L1724H | 1 | 0 | 2.240 | 99 | deleterious | Proline rich region | Sato *et al*. (2012) |
|  | 22 | g.51169830C>T | p.G1796E | 7 | 0 | 2.350 | 98 | deleterious | Proline rich region | Sato *et al*. (2012) |
|  | 23 | g.51165932C>T | p.D1926N | 2 | 0 | 3.670 | 23 | neutral | Proline rich region | Sato *et al*. (2012) |
|  | 23 | g.51165929C>T | p.D1927N | 1 | 0 | 3.670 | 23 | deleterious | Proline rich region | Sato *et al*. (2012) |
|  | 23 | g.51165767G>A | p.R1981C | 1 | 0 | 2.890 | 180 | deleterious | Proline rich region | Sato *et al*. (2012) |
|  | 23 | g.51165632C>T | p.G2026R | 1 | 0 | 3.460 | 125 | neutral | Proline rich region | This study |
|  | 23 | g.51165574C>A | p.G2045V | 1 | 0 | 3.430 | 109 | deleterious | - | Sato *et al*. (2012) |
| ASD & Controls | 22 | g.51172526G>C rs41275782 | p.D897E | 48, MAF>1%, P=0.05 | 17, MAF>1% | -2.550 | 45 | neutral | - | Sato *et al*. (2012), this study |
|  | 22 | g.51170706T>C rs3745521 | p.V1504A | 557, MAF>1%, P=0.71 | 447, MAF>1% | -0.142 | 64 | neutral | Proline rich region | Sato *et al*. (2012), this study |
|  | 22 | g.51169797G>A | p.P1807L | 4 | 8 | 1.120 | 98 | neutral | Proline rich region | Sato *et al*. (2012), this study |
|  | 23 | g.51165775G>A rs117056219 | p.T1978M | 19, MAF>1%, P=0.71 | 14, MAF>1% | 2.910 | 81 | neutral | Proline rich region | Sato *et al*. (2012), this study |
| Controls only | 1 | g.51219987C>T | p.V64I | 0 | 1 | 3.210 | 29 | deleterious | SPN domain | This study |
|  | 10 | g.51207049G>A | p.R421W | 0 | 1 | 3.890 | 101 | deleterious | - | This study |
|  | 22 | g.51170692C>T | p.G1509S | 0 | 1 | 1.780 | 56 | neutral | Proline rich region | This study |
|  | 22 | g.51169776C>G | p.G1814A | 0 | 1 | -1.260 | 60 | neutral | Proline rich region | This study |
|  | 22 | g.51169713C>A | p.R1835L | 0 | 1 | 2.590 | 102 | deleterious | Proline rich region | This study |
|  | 23 | g.51165601C>T rs144719481 | p.R2036H | 0 | 1 | 3.460 | 29 | deleterious | - | This study |
|  | 23 | g.51165266G>A | p.R2148S | 0 | 1 | 1.240 | 110 | deleterious | SAM domain | This study |
