# Supplementary material for: Meta-analysis of SHANK Mutations in Autism Spectrum Disorders: A Gradient of Severity in Cognitive Impairments
Source: PLoS Genet. 2014 Sep 4;10(9):e1004580. doi: 10.1371/journal.pgen.1004580 (PMC4154644; doi:10.1371/journal.pgen.1004580)
Supplement: Table S10 — SHANK2 coding-sequence variants identified in 851 patients with ASD and 1 090 controls. #Indicates de novo mutations. aNucleotide positions are according to NM_012309.3 from NCBI37/hg19 on the positive DNA strand (chromosome 11). bMaximum Grantham score (215) given for non-sense variants. The patients with ASD and the controls used for this analysis came from Leblond et al. (2012) (455 ASD & 431 controls) and from the study of Berkel et al. (2010) (396 ASD & 659 controls). The Grantham matrix and GERP scores were obtained from SeattleSeq Annotation 134. We used the Fisher's exact test (2-sided) and Pearson's Chi-squared test with Yates' continuity correction. P, p-value; ASD, Autism Spectrum Disorder; MAF, Minor Allele Frequency; GERP, Genomic Evolutionary Rate Profiling; pph2_class, polyphen-2_class. (DOC) [file pgen.1004580.s016.doc]

Table S10: *SHANK2* coding-sequence variants identified in 851 patients with ASD and 1 090 controls

|  | Detected variants | | | Frequency | | GERP | Grantham | pph2_class | Protein domain | Study |
| --- | --- | --- | --- | --- | --- | --- | --- | --- | --- | --- |
|  | Exon | Nucleotide/ dbSNP^a^ | Amino acid | ASD  (n=851) | Controls (n=1 090) |  |  | Hum_Div |  |  |
| ASD only | 11 | g.70666749G>A | p.R405W | 1 | 0 | 3.460 | 101 | deleterious | - | Berkel *et al*. (2010) |
|  | 11 | g.70666635G>A | p.R443C | 1 | 0 | 4.190 | 180 | deleterious | - | Leblond *et al*. (2012) |
|  | 13 | g.70644566G>A rs149996975 | p.P587S | 1 | 0 | 4.440 | 74 | deleterious | - | Berkel *et al*. (2010) |
|  | 14 | g.70544853C>A | p.R598L | 1 | 0 | 4.740 | 102 | deleterious | - | Leblond *et al*. (2012) |
|  | 17 | g.70348949C>A | p.V717F | 1 | 0 | 4.460 | 50 | deleterious | PDZ domain | Leblond *et al*. (2012) |
|  | 17 | g.70348913C>T | p.A729T | 1 | 0 | 3.540 | 58 | deleterious | - | Leblond *et al*. (2012) |
|  | 22 | g.70336411G>A | p.R841X^#^ | 1 | 0 | 4.630 | 215^b^ | deleterious | Proline rich region | Berkel *et al*. (2010) |
|  | 24 | g.70332914C>T | p.E1162K | 1 | 0 | 4.130 | 56 | deleterious | Proline rich region | Leblond *et al*. (2012) |
|  | 24 | g.70332890C>T rs143671037 | p.G1170R | 1 | 0 | 3.130 | 125 | deleterious | Proline rich region | Leblond *et al*. (2012) |
|  | 24 | g.70332272C>T | p.V1376I | 1 | 0 | 5.060 | 29 | deleterious | Proline rich region | Leblond *et al*. (2012) |
|  | 24 | g.70332239-70332234dupTTGCCA | p.L1387-P1388dup | 1 | 0 | - | - | deleterious | Proline rich region | Berkel *et al*. (2010) |
|  | 24 | g.70331881G>A rs146717159 | p.T1506M | 1 | 0 | 5.660 | 81 | deleterious | Proline rich region | Berkel *et al*. (2010) |
|  | 24 | g.70331795C>T | p.D1535N | 1 | 0 | 5.420 | 23 | deleterious | Proline rich region | Leblond *et al*. (2012) |
|  | 25 | g.70319359A>G | p.L1722P | 1 | 0 | 3.620 | 98 | deleterious | Proline rich region | Leblond *et al*. (2012) |
|  | 25 | g.70319339C>T | p.A1729T | 1 | 0 | 1.500 | 58 | neutral | Proline rich region | Berkel *et al*. (2010) |
| ASD & Controls | 11 | g.70666733G>A | p.T410M | 1 | 2 | 4.380 | 81 | deleterious | - | Leblond *et al*. (2012), Berkel *et al*. (2010) |
|  | 13 | g.70644655C>T rs141184740 | p.S557N | 9, P=0.039, OR=3.9 [1-22] | 3 | 4.440 | 46 | deleterious | SH3 domain | Leblond *et al*. (2012), Berkel *et al*. (2010) |
|  | 13 | g.70322267C>T | p.R569H | 17, MAF>1%, P=0.50 | 28, MAF>1% | 4.440 | 29 | deleterious | SH3 domain | Leblond *et al*. (2012), Berkel *et al*. (2010) |
|  | 21 | g.70338541T>G rs55968949 | p.K780Q | 4 | 4 | 4.530 | 53 | deleterious | Proline rich region | Leblond *et al*. (2012), Berkel *et al*. (2010) |
|  | 22 | g.70336479C>T rs117843717 | p.R818H | 8 | 7 | 4.630 | 29 | deleterious | Proline rich region | Leblond *et al*. (2012), Berkel *et al*. (2010) |
|  | 24 | T70011146C rs62622853 | p.Y967C | 27, MAF>1%, P=0.8 | 38, MAF>1% | 3.730 | 194 | deleterious | Proline rich region | Leblond *et al*. (2012), Berkel *et al*. (2010) |
|  | 24 | g.70331641G>A rs141276059 | p.P1586L | 4 | 1 | 5.420 | 98 | deleterious | Proline rich region | Leblond *et al*. (2012), Berkel *et al*. (2010) |
|  | 25 | g.70319373C>T rs140134890 | p.M1717I | 2 | 2 | 0.461 | 10 | neutral | Proline rich region | Leblond *et al*. (2012), Berkel *et al*. (2010) |
| Controls only | 11 | g.70666719G>A | p.R415W | 0 | 1 | 3.430 | 101 | deleterious | - | Berkel *et al*. (2010) |
|  | 11 | g.70666668C>T | p.D432N | 0 | 1 | 4.380 | 23 | neutral | - | Berkel *et al*. (2010) |
|  | 11 | g.70666605T>C | p.M453V | 0 | 1 | -5.280 | 21 | neutral | - | Leblond *et al*. (2012) |
|  | 12 | g.70653229T>C | p.E514G | 0 | 1 | 3.320 | 98 | deleterious | - | Berkel *et al*. (2010) |
|  | 15 | g.70507751A>G | p.L629P | 0 | 1 | 4.760 | 98 | deleterious | PDZ domain | Leblond *et al*. (2012) |
|  | 21 | g.70338492G>T | p.T796N | 0 | 1 | 4.530 | 65 | deleterious | Proline rich region | Berkel *et al*. (2010) |
|  | 21 | g.70338478G>T | p.P801T | 0 | 1 | 4.530 | 38 | deleterious | Proline rich region | Berkel *et al*. (2010) |
|  | 22 | g.70336468C>T | p.A822T | 0 | 1 | 4.630 | 58 | deleterious | Proline rich region | Leblond *et al*. (2012) |
|  | 22 | g.70336465C>T | p.V823M | 0 | 1 | 3.680 | 21 | deleterious | Proline rich region | Leblond *et al*. (2012) |
|  | 22 | g.70336458G>A | p.T825M | 0 | 1 | 4.630 | 81 | deleterious | Proline rich region | Berkel *et al*. (2010) |
|  | 24 | g.70332530G>A | p.R1290W | 0 | 1 | 2.180 | 101 | deleterious | Proline rich region | Leblond *et al*. (2012) |
|  | 24 | g.70332475T>C | p.Q1308R | 0 | 1 | 2.990 | 43 | neutral | Proline rich region | Leblond *et al*. (2012) |
|  | 24 | g.70332299G>C | p.P1367A | 0 | 1 | 3.930 | 27 | deleterious | Proline rich region | Leblond *et al*. (2012) |
|  | 24 | g.70332032G>T | p.P1456T | 0 | 1 | 3.220 | 38 | deleterious | Proline rich region | Berkel *et al*. (2010) |
|  | 25 | g.70319533A>G rs150857128 | p.I1664T | 0 | 2 | 4.780 | 89 | neutral | Proline rich region | Berkel *et al*. (2010) |
|  | 25 | g.70319438C>T | p.D1696N | 0 | 1 | 5.910 | 23 | deleterious | Proline rich region | Berkel *et al*. (2010) |
