# Supplementary material for: Meta-analysis of SHANK Mutations in Autism Spectrum Disorders: A Gradient of Severity in Cognitive Impairments
Source: PLoS Genet. 2014 Sep 4;10(9):e1004580. doi: 10.1371/journal.pgen.1004580 (PMC4154644; doi:10.1371/journal.pgen.1004580)
Supplement: Table S11 — SHANK3 coding-sequence variants identified in 2 147 patients with ASD and 1 031 controls. #Indicates de novo mutations. +Q1243X was identified during an additional screen (nASD = 138) of exon 21 of SHANK3 and was not included in the meta-analysis. aNucleotide positions are according to NM_033517 from NCBI37/hg19 on the positive DNA strand (chromosome 22); bFor the variants with MAF>1%, the frequency was assessed only in PARIS cohort. cAverage GERP score for two sites flanking the insertion or average GERP score for deleted nucleotides; dMaximum Grantham score (215) given for splice, non-sense and frameshifting variants. The patients with ASD and the controls used for this analysis came from this study (n = 429) and from the studies reported in Table S5. The Grantham matrix and GERP scores were obtained from SeattleSeq Annotation 134. We used the Fisher's exact test (2-sided) and Pearson's Chi-squared test with Yates' continuity correction. P, p-value; ASD, Autism Spectrum Disorder; MAF, Minor Allele Frequency; GERP, Genomic Evolutionary Rate Profiling, pph2_class, polyphen-2_class. (DOC) [file pgen.1004580.s017.doc]

Table S11: *SHANK3* coding-sequence variants identified in 2 147 patients with ASD and 1 031 controls

|  | Detected variants | | | Frequency | | GERP | Grantham | pph2_class | Protein domain | Study |
| --- | --- | --- | --- | --- | --- | --- | --- | --- | --- | --- |
|  | Exon | Nucleotide/dbSNP^a^ | Amino acid | ASD  (n=2 147) | Controls (n=1 031) |  |  | HumDiv |  |  |
| ASD | 1 | g.51113103C>T | p.R12C | 2 | 0 | 0.869 | 180 | neutral | SPN domain | Durand *et al*. (2007) |
|  | 2 | g.51113615T>C | p.L68P | 1 | 0 | 3.910 | 98 | deleterious | SPN domain | Gauthier *et al*. (2008) |
|  | 4 | g.51117094C>G | p.P141A ^#^ | 1 | 0 | 4.830 | 27 | deleterious | - | Boccuto *et al*. (2012) |
|  | 5 | g.51117341C>G | p.A198G | 1 | 0 | 4.540 | 60 | neutral | Ank domain | Durand *et al*. (2007) |
|  | 8 | g.51121780C>T | p.R300C | 1 | 0 | 4.800 | 180 | deleterious | Ank domain | Durand *et al*. (2007) |
|  | 8 | g.51121844A>G | p.Q321R ^#^ | 1 | 0 | 4.800 | 43 | deleterious |  | Moessner *et al*. (2007) |
|  | 9 | g.51123071C>T | p.S341L | 1 | 0 | 4.070 | 145 | deleterious |  | Moessner *et al*. (2007) |
|  | 11 | g.51135995-51136006delCCCGGCCCCGCG | p.P442-A445del | 1 | 0 | - | - | deleterious |  | This study |
|  | 13 | g.51142357C>T | p.S561L | 1 | 0 | 5.420 | 145 | neutral |  | This study |
|  | 19 | g.51153464G>A | p.A752T | 1 | 0 | 4.210 | 58 | deleterious | Proline rich region | This study |
|  | I19 | g.51153476delG | IVS19+1g>- ^#^ | 1 | 0 | 4.210^c^ | 215^d^ | deleterious | Proline rich region | Gauthier *et al*. (2008) |
|  | 21a | g.51158686G>T | p.E809X ^#^ | 1 | 0 | 4.560 | 215^d^ | deleterious | Proline rich region | This study |
|  | 21a | g.51158945T>C | p.L895P | 1 | 0 | 4.560 | 98 | deleterious | Proline rich region | This study |
|  | 21a | g.51159169G>T | p.A970S | 1 | 0 | 4.420 | 99 | deleterious | Proline rich region | Moessner *et al*. (2007) |
|  | 21a | g.51159275delC | p.P1005Rfs*73 | 1 | 0 | 2.050^c^ | 215^d^ | deleterious | Proline rich region | This study |
|  | 21a | g.51159293G>T | p.G1011V | 4 | 0 | 3.020 | 109 | deleterious | Proline rich region | This study, Durand *et al*. (2007) |
|  | 21a | g.51159458G>T | p.R1066L | 1 | 0 | 3.720 | 102 | deleterious | Proline rich region | Durand *et al*. (2007) |
|  | 21a | g.51159488-51159492delCCCTG | p.A1076Efs*218 ^#^ | 1 | 0 | 2.912^c^ | 215^d^ | deleterious | Proline rich region | This study |
|  | 21a | g.51159685-51159686delCT | p.L1142Vfs*153 ^#^ | 1 | 0 | 0.942^c^ | 215^d^ | deleterious | Proline rich region | This study |
|  | 21a | g.51159778G>A RS139686326 | p.A1173T | 1 | 0 | 4.300 | 58 | neutral | Proline rich region | Moessner *et al*. (2007) |
|  | 21a | g.51159884G>A | p.G1208D | 1 | 0 | 4.410 | 94 | deleterious | Proline rich region | Schaaf *et al*. (2011) |
|  | 21a | g.51160049C>T | p.P1263L | 1 | 0 | 2.640 | 98 | deleterious | Proline rich region | Moessner *et al*. (2007) |
|  | 21a | g.51159866-51159869delCCCT | p.S1202Cfs*81 ^#^ | 1 | 0 | 3.155^c^ | 215^d^ | deleterious | Proline rich region | This study |
|  | 21a | g.51159940-51159941insG | p.A1227Gfs*69 | 1 | 0 | 2.840^c^ | 215^d^ | deleterious | Proline rich region | Durand *et al*. (2007) |
|  | 21a | g.51159953G>A | p.R1231H | 1 | 0 | -2.240 | 29 | neutral | Proline rich region | Durand *et al*. (2007) |
|  | 21a | g. 51159965C>A | p.P1235Q | 1 | 0 | 3.520 | 76 | deleterious | Proline rich region | This study |
|  | 21a | g.51159988C>T | p.Q1243X^+^ | 1 | 0 | 3.710 | 215^d^ | deleterious | Proline rich region | This study |
|  | 21a | g.51160018A>T | p.S1253C | 2 | 0 | 3.520 | 112 | neutral | Proline rich region | Schaaf *et al*. (2011) |
|  | 21a | g.51160025-51160037delGGGCCCAGCCCCC | p.R1255Lfs*25 ^#^ | 1 | 0 | 2,893^c^ | 215^d^ | deleterious | Proline rich region | This study |
|  | 21a | g. 51160057G>A | p.A1266T | 1 | 0 | 0.020 | 58 | neutral | Proline rich region | This study |
|  | 21a | g.51160070-51160071insGGCCA | p.G1271Afs*15 ^#^ | 1 | 0 | 4.515^c^ | 215^d^ | deleterious | Proline rich region | This study |
|  | 21a | g.51160086A>T | p.E1275D | 1 | 0 | 1.220 | 45 | deleterious | Proline rich region | This study |
|  | 21a | g.51160144delG | p.E1295Rfs*90 | 1 | 0 | 4.800 | 215^d^ | deleterious | Proline rich region | Boccuto *et al*. (2012) |
|  | 21a | g.51160275-51160276delAG | p.G1339Efs*5 | 1 | 0 | 4.310^c^ | 215^d^ | deleterious | Proline rich region | This study |
|  | 21a | g.51160477C>G | p.L1406V | 1 | 0 | 4.200 | 32 | neutral | Proline rich region | Moessner *et al*. (2007) |
|  | 21a | g.51160589T>C | p.M1443T | 1 | 0 | 4.260 | 81 | neutral | Proline rich region | Moessner *et al*. (2007) |
|  | 21a | g.51160615G>T | p.A1452S | 1 | 0 | 3.220 | 99 | neutral | Proline rich region | Boccuto *et al*. (2012) |
|  | 21a | g.51160618-51160634delGGCTGGCCTCTGCCG | p.G1453-A1457del | 1 | 0 | - | - | deleterious | Proline rich region | Moessner *et al*. (2007) |
|  | 22 | g.51169207C>T | p.P1555S | 1 | 0 | 3.950 | 74 | deleterious | Proline rich region | Schaaf *et al*. (2011) |
|  | 22 | g.51169213G>A | p.G1557S | 1 | 0 | 1.670 | 56 | neutral | Proline rich region | Moessner *et al*. (2007) |
|  | 22 | g.51169240A>G | p.S1566G | 1 | 0 | -0.766 | 56 | neutral | Proline rich region | Durand *et al*. (2007) |
|  | 22 | g.51169259C>T | p.A1572V | 2 | 0 | 3.910 | 64 | deleterious | Proline rich region | Schaaf *et al*. (2011) |
|  | 22 | g.51169364C>T | p.T1607I | 1 | 0 | 2.760 | 89 | deleterious | Proline rich region | Schaaf *et al*. (2011) |
|  | 22 | g.51169442G>A | p.R1633H | 1 | 0 | 4.010 | 29 | deleterious | - | Schaaf *et al*. (2011) |
|  | 22 | g.51169459C>T | p.P1639S | 1 | 0 | 2.860 | 74 | neutral | - | Schaaf *et al*. (2011) |
|  | 22 | g.51169463C>T | p.S1640L | 1 | 0 | 1.740 | 145 | deleterious | - | Schaaf *et al*. (2011) |
|  | 22 | g.51169480G>A | p.A1646T | 1 | 0 | 0.409 | 58 | neutral | - | Schaaf *et al*. (2011) |
|  | 22 | g.51169499G>A | p.G1652D | 3 | 0 | 2.840 | 94 | neutral | - | Schaaf *et al*. (2011) |
| ASD & Controls | 6 | g.51117516G>A | p.A224T | 1 | 1 | 5.180 | 58 | deleterious | - | Durand *et al*. (2007), Gauthier *et al*. (2008) |
|  | 6 | g.51117580T>C rs9616915 | p.I245T | 205/270^b^, MAF>1%, P=0.9 | 117/181^b^, MAF>1% | 4.650 | 89 | neutral | - | All |
|  | 19 | g.51153371G>A rs61729471 | p.A721T | 14/200^b^, MAF>1%, P=0.2 | 6/174^b^ , MAF>1% | 4.060 | 58 | deleterious | - | All |
|  | 21a | g.51158975C>G | p.A905G | 2 | 1 | -8.590 | 60 | neutral | Proline rich region | This study |
|  | 21a | g.51160154G>A | p.R1298K | 2 | 2 | 4.800 | 26 | deleterious | Proline rich region | Gauthier *et al*. (2010), Schaaf *et al*. (2011) |
|  | 21a | g.51160231G>A | p.A1324T | 1 | 1 | -0.031 | 58 | neutral | Proline rich region | Gauthier *et al*. (2008), Schaaf *et al*. (2011) |
|  | 21a | g.51160259T>G | p.V1333G | 2 | 5 | 1.220 | 109 | neutral | Proline rich region | This study, Durand *et al*. (2007), Gauthier *et al*. (2010), Schaaf *et al*. (2011) |
|  | 22 | g.51169504C>A | p.P1654T | 9 | 9 | 1.120 | 38 | neutral | - | This study, Durand *et al*. (2007), Schaaf *et al*. (2011), Moessner *et al*. (2007), Gauthier *et al*. (2008 & 2010) |
| Controls | 6 | g.51117489C>T | p.R215C | 0 | 1 | 4.160 | 180 | deleterious | - | Gauthier *et al*. (2008) |
|  | 16 | g.51143462G>A | p.R656H | 0 | 1 | 4.430 | 29 | deleterious | PDZ domain | Schaaf *et al*. (2011) |
|  | 21a | g.51160274C>T | p.S1338L | 0 | 1 | 4.930 | 145 | deleterious | Proline rich region | Gauthier *et al*. (2010) |
|  | 21a | g.51160394C>T | p.T1378M | 0 | 1 | 5.110 | 81 | deleterious | Proline rich region | Schaaf *et al*. (2011) |
|  | 21a | g.51160493C>T | p.P1411L | 0 | 1 | 5.290 | 98 | deleterious | Proline rich region | Schaaf *et al*. (2011) |
|  | 21a | g.51160540G>A | p.V1427M | 0 | 1 | 5.290 | 21 | deleterious | Proline rich region | Schaaf *et al*. (2011) |
|  | 22 | g.51169180A>G | p.I1546V | 0 | 2 | 3.950 | 29 | deleterious | Proline rich region | This study, Gauthier *et al*. (2008) |
|  | 22 | g.51169241G>T | p.S1566I | 0 | 1 | 3.950 | 142 | neutral | Proline rich region | Schaaf *et al*. (2011) |
|  | 22 | g.51169508G>A | p.G1655D | 0 | 1 | 3.860 | 94 | neutral | - | Schaaf *et al*. (2011) |
