# Supplementary material for: Meta-analysis of SHANK Mutations in Autism Spectrum Disorders: A Gradient of Severity in Cognitive Impairments
Source: PLoS Genet. 2014 Sep 4;10(9):e1004580. doi: 10.1371/journal.pgen.1004580 (PMC4154644; doi:10.1371/journal.pgen.1004580)
Supplement: Table S12 — Statistical power of the association between SHANK damaging missense variants and ASD. *Two-sided Fisher's exact test. (DOC) [file pgen.1004580.s018.doc]

Table S12: Statistical power of the association between *SHANK* damaging missense variants and ASD

|  | Sample size | | Frequency of the variants (%) | |  |  |  | Power to detect | | |
| --- | --- | --- | --- | --- | --- | --- | --- | --- | --- | --- |
|  | Patients | Controls | Patients | Controls | Odd Ratio | P* | Achieved power | Odd Ratio=1.5 | Odd Ratio=2 | Odd Ratio=3 |
| SHANK1 damaging missense | 760 | 492 | 3.16 | 1.02 | 3.17 | 0.012 | 69% | 8% | 23% | 65% |
| SHANK2 damaging missense | 851 | 1090 | 4.58 | 2.66 | 1.76 | 0.025 | 59% | 34% | 85% | 99% |
| SHANK3 damaging missense | 2147 | 1031 | 1.3 | 1.07 | 1.22 | 0.73 | 8% | 17% | 51% | 96% |
