# Supplementary material for: Meta-analysis of SHANK Mutations in Autism Spectrum Disorders: A Gradient of Severity in Cognitive Impairments
Source: PLoS Genet. 2014 Sep 4;10(9):e1004580. doi: 10.1371/journal.pgen.1004580 (PMC4154644; doi:10.1371/journal.pgen.1004580)
Supplement: Text S1 — Supplementary method describing the genomic structure of the SHANK genes, coverage of SHANK genes by exome sequencing, cytogenetic analysis and FISH, multiplex ligation-dependent probe amplification (MLPA), genetic ancestry of patients with ASD and controls. (DOC) [file pgen.1004580.s019.doc]

**Meta-analysis of *SHANK* mutations in Autism Spectrum Disorders:**

**A gradient of severity in cognitive impairments**

**Text S1**

Claire S Leblond^1,2,3^, Caroline Nava^4,5,6^, Anne Polge^7^, Julie Gauthier^8^, Guillaume Huguet^1,2,3^, Serge Lumbroso^7^, Fabienne Giuliano^9^, Coline Stordeur^1,2,3,13^, Christel Depienne^4,5,6^, Kevin Mouzat^7^, Dalila Pinto^10^, Jennifer Howe^11^, Nathalie Lemière^1,2,3^, Christelle M Durand^1,2,3^, Jessica Guibert^1,2,3^, Elodie Ey^1,2,3^, Roberto Toro^1,2,3^, Hugo Peyre^12^, Alexandre Mathieu^1,2,3^, Frédérique Amsellem^1,13,14^, Maria Rastam^15^, I. Carina Gillberg^16^, Gudrun A Rappold^17^, Richard Holt^18^, Anthony P Monaco^18^, Elena Maestrini^19^, Pilar Galan^20^, Delphine Heron^21,22,23^, Aurélia Jacquette^21,22^, Alexandra Afenjar^21,22,23^, Agnès Rastetter^4,5,6^, Alexis Brice^4,5,6^, Françoise Devillard^24^, Brigitte Assouline^25^, Fanny Laffargue^26^, James Lespinasse^27^, Jean Chiesa^28^, François Rivier^29^, Dominique Bonneau^30,31^, Beatrice Regnault^32^, Diana Zelenika^33^, Marc Delepine^33^, Mark Lathrop^33^, Damien Sanlaville^34^, Caroline Schluth-Bolard^34^, Patrick Edery^34^, Laurence Perrin^35^, Anne Claude Tabet^35^, Michael J Schmeisser^36^, Tobias M Boeckers^36^, Mary Coleman^37^, Daisuke Sato^11^, Peter Szatmari^11^, Stephen W Scherer^11^, Guy A Rouleau^38^, Catalina Betancur^5,39,40^, Marion Leboyer^14,41,42,43^, Christopher Gillberg^16,44^, Richard Delorme^1,2,3,13,14$^, Thomas Bourgeron^1,2,3,14$*^

Genomic structure of the *SHANK* genes

There are three human *SHANK* genes and the topology of the SHANK protein phylogenetic tree indicates that the gene duplication giving rise to *SHANK2* and *SHANK3* occurred after the *SHANK1* split (Figure S1). The encoded proteins contain ankyrin domains (ANK), one SH3 (Src Homology 3) domain, one PDZ (PSD95/DLG/ZO1) domain and one SAM (Sterile Alpha Motif) domain. For each *SHANK* gene, short and long isoforms exist due to the presence of alternative promoters and exons [58,59]. *SHANK1* is located at 19q13.33 and spans 55.1 kb. The gene contains 23 exons and alternative promoters leading to two different isoforms. The long one, *SHANK1A,* includes all the above-mentioned protein domains, whereas the short one, *SHANK1B,* only contains the PDZ and SAM domains. *SHANK2* is located at 11q13.3 and spans 621.8 kb. It contains 25 exons, three different promoters and alternative stop codons leading to four different isoforms: the largest one, *SHANK2E,* that contains all the domains; *ProSAP1A,* starting before the SH3 domain; *ProSAP1,* starting before the PDZ domain; and *AF141901,* ending before the SAM domain [18]. *SHANK3* is located at 22q13.3 and spans 55.1 kb. It contains 24 exons and alternative stop codons located in exon 21b and 24 leading to at least two different isoforms [6]. In mice, a long isoform ( containing all protein domains), a medium isoform (β, starting before the SH3 domain), and a short isoform , starting before the PDZ domain of Shank3/ProSAP2 have been described [28,58,60]. Conserved CpG islands between the mouse and human *SHANK3* have been described, suggesting the existence of these isoforms also in humans [61-63].

The genomic sequence covering exons 8 and 9 of the human *SHANK2* is missing in the GRCh37/hg19 assembly of the human genome. The surrounding sequences of these two exons were obtained using a standard nucleotide Basic Local Alignment Search Tool (BLAST, <http://blast.ncbi.nlm.nih.gov/Blast.cgi>). We first aligned the *AB208025* mRNA (containing exons 8 and 9) and the nucleotide collection (nr/nt) database. A perfect sequence match was observed with the *AC234421.2* sequence from GeneBank (Homo sapiens FOSMID clone ABC9-43854600O13). Intronic sequences (Table S1) were deduced from the alignment of *AB208025* and *AC234421.2* sequences, and validated by PCR and sequencing. Amplification of these exons in 180 individuals from the human genome diversity panel (HGDP) confirmed that these exons were present in all populations (data not shown).

*SHANK3* was previously represented by the *NM_001080420* sequence that contained two major errors, specifically the inclusion of an unsupported exon (wrongly annotated as exon 11) and a sequence error at the 5’ end of the real exon 11 (Figure S2). These errors led to false interpretations of the results of *SHANK3* screening in patients with ASD. Kolevzon *et al*. (2011) and Boccuto *et al*. (2012) described a G insertion located in exon 11 of *NM*_*001080420* in two unrelated patients with ASD [17,64]. As pointed out by Kolevzon *et al*. (2011) [64], exon 11 of *NM*_*001080420* is non-coding (absent in the human mRNA AB569469 or ESTs sequences from GenBank), so the G insertion is actually located in intron 10. The genomic sequence covering the real exon 11 was missing in the GRCh37/hg19 assembly of the human genome. We obtained the surrounding sequences of exon 11 by a standard BLAST procedure using the *AB5694695* mRNA sequence (the coding sequence of the real exon 11) and the nucleotide collection (nr/nt) database. A perfect sequence match was observed with *AC194790.3* from GeneBank (*Pan troglodytes* BAC clone CH251-692N12). Intronic sequences (Table S1) were deduced from the alignment between *AB5694695* and *AC194790.3* sequences, and then validated by PCR and sequencing. We submitted the genomic sequence covering exons 8 and 9 of *SHANK2* (BankIt1539237; sequences JX122808 & JX122809) and exon 11 of *SHANK3* (BankIt1539238; sequence JX122810) to the GenBank.

Coverage of *SHANK* genes by exome sequencing

We ascertained the average sequencing coverage of each *SHANK* gene using exome and whole genome sequencing. For exome sequencing, we used data from 3510 individuals downloaded from the Exome Variant Server of NHLBI Exome Sequencing Project, Seattle, WA (<http://evs.gs.washington.edu/EVS/>). For whole genome sequencing, we used 54 individual genomes sequenced by Complete Genomics ([www.completegenomics.com](http://www.completegenomics.com/)). For both methods, we observed a very low sequencing coverage of *SHANK1* and *SHANK3* (Figure S3). Whole genome sequencing seems to be a better approach than whole exome sequencing, but none of these technologies covered the entire coding sequence of *SHANK* genes. For example, a 10x coverage is achieved for only 74.1% and 50.4% of *SHANK3* with whole genome sequencing and whole exome sequencing, respectively. As a consequence, until more progress is made, Sanger sequencing remains the method of choice to search for mutations in *SHANK1* and *SHANK3*.

Cytogenetic analysis and FISH

High-resolution karyotypes were performed on peripheral blood lymphocytes using RHG (R-bands by heating using Giemsa) and GTG (G-bands by trypsin using Giemsa) banding. FISH using the “RP11-102B19” probe covering *SHANK2* was performed on metaphase spreads of RDB_30769 individuals (RP11-102B19, BlueFish, BlueGnome, Cambridge, UK). Both chromosomes 11 are recognizable using the « reverse DAPI » tool (Genikon software, Alphelys). FISH, Fluorescent In Situ Hybridization.

Multiplex Ligation-dependent Probe Amplification (MLPA)

The MLPA assay was performed using 100 ng of genomic DNA and three independent probemix (P188-B2, P343-C1 and P339-A1) as recommended by the manufacturer (MRC Holland, Amsterdam, The Netherlands). The probemix contain probes along the locus 22q13 and more particularly covering different exons of *SHANK3* (Figure S4). The MLPA fragments were studied on a 3130xl Genetic Analyser (Applied Biosystems, France). The results were analyzed with the GeneMapper v4.0 (Applied Biosystems, USA) and the Coffalyzer v7 softwares (MRC Holland).

Genetic ancestry of patients with ASD and controls

Multidimensional scaling (MDS) analysis using linkage clustering of individuals, and based on genome-wide pairwise identity-by-state distance, was performed in order to assess the genetic ancestry of the individuals (n=430 ASD and n=837 controls) of PARIS and SUVIMAX cohorts. We used PLINK 1.90 beta (<https://www.cog-genomics.org/plink2/>) and the function “--cluster & --mds-plot”. The genotyping data of PARIS and SUVIMAX cohorts were obtained using Illumina Human BeadChip (1M-Duo, Omni 1 and Omni 2.5). The genotyping data from the International HapMap project (<http://hapmap.ncbi.nlm.nih.gov/>) including individuals with European (CEU & TSI), Asian (JPT, CHB & CHD), African (ASW, LWK, MKK & YRI), Mexican (MEX) and Indian (GIH) ancestry were used to calculate the genetic distance between individuals (Figure S6). PARIS, Paris Autism Research International Sibpair; ASD, Autism Spectrum Disorder; SUVIMAX, “Supplémentation en Vitamines et Minéraux Antioxydants”. ASW, African ancestry in Southwest USA; CEU, Utah residents with Northern and Western European ancestry from the CEPH collection; CHB, Han Chinese in Beijing (China); CHD, Chinese in Metropolitan Denver (Colorado); GIH, Gujarati Indians in Houston (Texas); JPT, Japanese in Tokyo (Japan); LWK, Luhya in Webuye (Kenya); MXL, Mexican ancestry in Los Angeles (California); MKK, Maasai in Kinyawa (Kenya); TSI, Toscani in Italia; YRI, Yoruba in Ibadan (Nigeria).

References

58. Boeckers TM, Bockmann J, Kreutz MR, Gundelfinger ED (2002) ProSAP/Shank proteins - a family of higher order organizing molecules of the postsynaptic density with an emerging role in human neurological disease. J Neurochem 81: 903-910.

59. Kreienkamp HJ (2008) Scaffolding proteins at the postsynaptic density: shank as the architectural framework. Handb Exp Pharmacol: 365-380.

60. Uchino S, Waga C (2013) SHANK3 as an autism spectrum disorder-associated gene. Brain Dev 35: 106-110.

61. Ching TT, Maunakea AK, Jun P, Hong C, Zardo G, et al. (2005) Epigenome analyses using BAC microarrays identify evolutionary conservation of tissue-specific methylation of SHANK3. Nat Genet 37: 645-651.

62. Beri S, Tonna N, Menozzi G, Bonaglia MC, Sala C, et al. (2007) DNA methylation regulates tissue-specific expression of Shank3. J Neurochem 101: 1380-1391.

63. Maunakea AK, Nagarajan RP, Bilenky M, Ballinger TJ, D'Souza C, et al. (2010) Conserved role of intragenic DNA methylation in regulating alternative promoters. Nature 466: 253-257.

64. Kolevzon A, Cai G, Soorya L, Takahashi N, Grodberg D, et al. (2011) Analysis of a purported SHANK3 mutation in a boy with autism: clinical impact of rare variant research in neurodevelopmental disabilities. Brain Res 1380: 98-105.
